# Optimized transgene expression in the red alga *Porphyridium purpureum* and efficient recombinant protein secretion into the culture medium

**DOI:** 10.1007/s11103-024-01415-2

**Published:** 2024-02-14

**Authors:** Alexander Hammel, Juliane Neupert, Ralph Bock

**Affiliations:** Max-Planck-Institut für Molekulare Pflanzenphysiologie (MPI-MP), Am Mühlenberg 1, 14476 Potsdam-Golm, Germany

**Keywords:** Codon usage, GC content, Translation, RNA stability, Protein secretion, Molecular farming

## Abstract

Microalgae represent a promising but yet underexplored production platform for biotechnology. The vast majority of studies on recombinant protein expression in algae have been conducted in a single species, the green alga *Chlamydomonas reinhardtii*. However, due to epigenetic silencing, transgene expression in *Chlamydomonas* is often inefficient. Here we have investigated parameters that govern efficient transgene expression in the red microalga *Porphyridium purpureum*. *Porphyridium* is unique in that the introduced transformation vectors are episomally maintained as autonomously replicating plasmids in the nucleus. We show that full codon optimization to the preferred codon usage in the *Porphyridium* genome confers superior transgene expression, not only at the level of protein accumulation, but also at the level of mRNA accumulation, indicating that high translation rates increase mRNA stability. Our optimized expression constructs resulted in YFP accumulation to unprecedented levels of up to 5% of the total soluble protein. We also designed expression cassettes that target foreign proteins to the secretory pathway and lead to efficient protein secretion into the culture medium, thus simplifying recombinant protein harvest and purification. Our study paves the way to the exploration of red microalgae as expression hosts in molecular farming for recombinant proteins and metabolites.

**Supplementary Information:**

The online version contains supplementary material available at 10.1007/s11103-024-01415-2.

## Key message

This study determines the impact of codon usage on mRNA stability and protein accumulation in a red alga, and demonstrates the possibility to harvest recombinant proteins from the culture medium.

**Supplementary Information:**

The online version contains supplementary material available at 10.1007/s11103-024-01415-2.

## Introduction

Microalgae have long been considered as excellent production systems for recombinant proteins, high-value metabolites, biofuels and feedstocks for the chemical industry (Walker et al. 2005; Hu et al. 2008; Rosenberg et al. 2008; Rosales-Mendoza et al. 2012; Gangl et al. 2015). Their photoautotrophic growth and their potential to avoid competition with food and feed production for arable land makes them particularly attractive. To date, the vast majority of studies on the biotechnological exploitation of algae has been conducted in a single model species, the unicellular green alga *Chlamydomonas reinhardtii* (reviewed, e.g., in Scaife et al. 2015). However, the relatively low cell densities that *Chlamydomonas* can be grown to, the often poor expression of transgenes from the nuclear genome (Fuhrmann et al. 1999; Schroda et al. 2000) and the frequent loss of transgene expression over time (Cerutti et al. 1997; Yamasaki et al. 2008) have posed serious limitations to the use of the alga as a production host for proteinaceous biopharmaceuticals, industrial enzymes and other recombinant proteins of commercial value (Scaife et al. 2015). While stable transgene insertion into the nuclear genome is routine, the isolation of transgenic strains that express the foreign gene to reasonably high levels has been very challenging. The isolation of dedicated expression strains (Neupert et al. 2009) that are defective in epigenetic transgene silencing (Neupert et al. 2020) has partially alleviated the problem and, in recent years, facilitated the expression of a substantial number of transgenes (e.g., Lauersen et al. 2015; Barahimipour et al. 2016; Ramos-Martinez et al. 2017; Perozeni et al. 2020). However, expression levels usually remain lower than in seed plants, and the expression of long coding regions continues to be problematic and requires inclusion of intron sequences (Baier et al. 2018).

The exploitation of alternative algal species in biotechnology and synthetic biology has been largely hampered by the lack of molecular tools. Only very few algal species are currently robustly transformable and have been optimized for efficient expression of foreign genes. Especially, the large biodiversity of non-green algae currently remains nearly unexplored. Based on the few non-green algae that have been investigated (Fu et al. 2019), it seems likely that species can be identified that possess more favorable transgene expression properties and are more amenable to large-scale high cell-density fermentation than *Chlamydomonas*.

We have recently developed a genetic transformation method for the unicellular red alga *Porphyridium purpureum* (Li and Bock 2018). We discovered that transformation does not occur by integration of the transforming DNA into the nuclear genome. Instead, the transformation plasmids are episomally maintained and replicate in the algal nucleus to high copy numbers. At first sight, this seems surprising, given that the transformation vectors are based on bacterial plasmids that would not be expected to be capable of autonomous replication in the nucleus of a eukaryotic cell. However, plasmids have been found to naturally occur in certain strains of red algae (Goff and Coleman 1990; Lee et al. 2016), raising the possibility that they were horizontally acquired from bacterial donor organisms.

The extrachromosomal maintenance of the introduced foreign DNA at high copy number bodes well for transgene expression and, in particular, high-level recombinant protein production in *Porphyridium*. Here, we have explored determinants of efficient foreign protein expression in *Porphyridium*. We report a systematic comparison of the expression of a set of *YFP* gene variants that encode the identical amino acid sequence, but differ in GC content and codon usage. We show that full codon optimization (to the preferred codon usage in the *Porphyridium* nuclear genome) confers superior expression and leads to YFP accumulation to up to 5% of the total soluble protein (TSP). We also report new expression constructs that target foreign proteins to the secretory pathway and confer efficient protein secretion into the culture medium, opening up new possibilities for algal biotechnology and synthetic biology.

## Materials and methods

### Cultivation of microalgae

Cultivation of *Porphyridium purpureum* strain SAG 1380-1d (obtained from the Culture Collection of Algae at the University of Göttingen) was performed under photoautotrophic growth conditions at 25 °C, under agitation at 120 rpm and continuous light of 100 μmol photons m^−2^ s^−1^ (Li and Bock 2018) in low-salt artificial seawater (lsASW: 15.2 g/L NaCl, 4.9 g/L MgSO_4_, 2 g/L KNO_3_; Kathiresan et al. 2007). Cu1tivation of *Chlamydomonas reinhardtii* expression strain UVM11 (Neupert et al. 2009) was performed under standard mixotrophic growth conditions in TAP medium (Neupert et al. 2012).

### Determination of algal cell numbers

Cell numbers of algal strains grown in liquid culture were determined using the Z2 Coulter Particle Count and Size Analyzer (Beckman Coulter, USA). 100 µL cell culture were mixed with 9.9 mL counting buffer (Isoton® II Diluent, Beckman Coulter). Cell diameters from 3.5 µm to 12 µm were considered for cell number determination. Cells were counted twice and the average value was used.

### Codon optimization and codon usage analysis

Codon optimization for *YFP* and codon usage analysis were performed as described previously (Barahimipour et al. 2015). To determine the codon usage of *P. purpureum,* 92 CDSs of the top highly expressed nucleus-encoded genes (comprising altogether 81,120 nt, i.e., 27,040 codons) were subjected to analysis with the webtool “codon usage” of the Sequence Manipulation Suite (bioinformatics.org/sms2/codon_usage). The codon usage table obtained was imported into the CodonWorkbench software (Dr. Marc Lohse, MPI-MP; http://www.buba-basis.de/software/cwb/cwb.html) for subsequent analysis and codon optimization.

### Construction of transformation vectors

All transformation vectors were constructed based on vector pZL22 (Li and Bock 2018) harboring the zeocin resistance gene *ble* under the control of the *Porphyridium purpureum* tubulin promoter and terminator. The chloroplast-targeted GFP was excised from pZL22 with the restriction enzymes XhoI and NheI, and substituted through Gibson assembly with *YFP* gene variants amplified from the previously described plasmids pRMB8b (cpYFP), pRMB11b (vYFP), pRMB13 (laYFP), and pRMB12 (CrYFP) (Barahimipour et al. 2015). The fully codon-optimized *PpYFP* was chemically synthesized (GeneCust) and cloned into a pBlueScript II vector. For promoter analysis, the actin promoter and the CDS in pZL22 were excised with the restriction enzymes SacII and NheI, and replaced via Gibson assembly with the new promoters (amplified from genomic DNA) and the *PpYFP* coding region (amplified from pASH1; Supplementary Table 3). For construction of transformation vectors conferring YFP secretion or ER targeting, a derivative of plasmid pASH8 (Supplementary Table 3) containing an SpeI restriction site positioned between the actin promoter and the CDS was constructed. The plasmid was then cut with SpeI and NheI, and the excised CDS was replaced via Gibson assembly with the carbonic anhydrase signal peptide amplified from genomic DNA and the PpYFP coding region (with or without the HDEL motif-encoding sequence) amplified from plasmid pASH1. All primers used are listed in Supplementary Table 3.

### Microscopy

Fluorescence of YFP was detected in living algal cells with a confocal laser-scanning microscope (TCS SP5; Leica, Wetzlar) using an argon laser for excitation (514 nm), a 510–535 nm filter for detection of YFP fluorescence, and a 630–720 nm filter for detection of chlorophyll fluorescence. For determination of the ratios of fluorescent to non-fluorescent algal cells, YFP and chlorophyll fluorescences were detected using the same filter with an epifluorescence BX-51 microscope (Olympus). Images were captured and automatically analyzed using the Image J software with a published macroscript (Grishagin 2015). The count of cells displaying chlorophyll fluorescence was considered as the total cell number, and compared to the count of cells showing YFP fluorescence. The ratio of fluorescent cells to total cell number was then calculated for each sample.

### Transformation of microalgae

Nuclear transformation of *P. purpureum* was performed using the biolistic method and a PDS-1000/He biolistic gun (Bio-Rad). 5 × 10^7^ cells from a culture in the exponential growth phase were harvested by centrifugation for 5 min at 4000 *g*, washed twice with lsASW medium, and plated on an lsASW agar plate without antibiotics. Bombardment was performed with 0.5 mg gold particles (0.6 µm diameter) coated with 1 µg undigested plasmid DNA using 1350 psi rupture discs from a distance of 9 cm. Afterwards, transformed cells were washed off the plate with lsASW, and washed twice in liquid medium to remove cell debris. Subsequently, the cells were plated on agar-solidified lsASW medium containing 25 mg/L zeocin, incubated in the dark overnight, and then transferred to the light at 100 µmol photons m^−2^ s^−1^ for 3 weeks. Emerging colonies were picked and transferred to liquid lsASW medium with 25 mg/L zeocin.

### Single colony isolation from *Porphyridium* cells

All attempts to obtain colonies from single cells of *Porphyridium* by plating a dilution series directly onto lsASW agar plates failed in that either no colonies (high dilutions), or a lawn of cells (low dilutions) grew on the plates. We then employed a new algal plating protocol (Skeffington et al. 2020) to obtain single cells. In this protocol, 200 µL of a diluted liquid culture of cells grown to the exponential phase are mixed with 1 mL of 0.5 g/mL wheat starch in lsASW, plated on agar-solidified lsASW medium, air dried, and incubated for two weeks at 100 µmol photons m^−2^ s^−1^ light intensity. Agar embedding of *P. purpureum* was performed by mixing 200 µL of a diluted liquid culture (of cells grown to the exponential phase) with 2.7 mL 0.4% agar-solidified lsASW. The suspension was plated on 1.5% agar-solidified lsASW plates, air dried, and incubated for 2 weeks at 100 µmol photons m^−2^ s^−1^ light intensity. Colonies obtained with both single-colony isolation methods were picked with a sterile toothpick and transferred into liquid lsASW medium (containing 50 µg/mL zeocin for cultivation of transgenic strains).

### Isolation of total cellular DNA from *Porphyridium*

Total genomic DNA was extracted from cultured *Porphyridium* cells using a modified CTAB-based method (Li and Bock 2018). Briefly, 5 mL samples of an algal culture in the exponential growth phase were harvested by centrifugation for 5 min at 4000 *g*. The pelleted cells were washed once with lsASW, then resuspended in 1 mL extraction buffer (2% cetyltrimethyl ammonium bromide, 100 mM Tris–HCl pH 8.0, 20 mM EDTA pH 8.0, 1.4 M NaCl, 2% polyvinylpyrrolidone (PVP) and 2% freshly added β-mercaptoethanol), and incubated with shaking at 65 °C for 30 min. Subsequently, 1 mL of phenol/chloroform/isoamyl alcohol (25:24:1) was added to the sample followed by thorough vortexing. The cell lysate was then centrifuged at 10 °C for 10 min at 12,000 *g*, and the upper (aqueous) phase was transferred to a new tube. RNA was removed by digestion with 10 µg RNase A for 30 min at 37 °C, followed by extraction with the same volume of chloroform/isoamyl alcohol (24:1). After centrifugation at 10 °C for 10 min at 12,000 *g*, the DNA was precipitated from the upper (aqueous) phase with 0.7 volumes of ice-cold isopropanol and incubation at − 20 °C for 1 h. The sample was then centrifuged at 4 °C for 10 min (at 17,000 *g*), the DNA pellet was washed with 70% ethanol (v/v), air-dried and resuspended in water.

### RNA extraction and northern blot analyses

Total RNA was isolated with the Trizol reagent (Thermo Fisher Scientific) according to the manufacturer’s protocol with some modifications. Briefly, 10 mL samples of cell cultures grown in liquid medium were harvested at the exponential growth phase by centrifugation at 2000 *g* at 4 °C for 2 min, and washed once with lsASW. The cell pellet was then resuspended in 2 mL Trizol and incubated for 5 min at 65 °C. Following homogenization by vortexing, cells were centrifuged at 12,000 *g* at 4 °C for 10 min, and the supernatant was collected. 400 µL chloroform were then added, the sample was vigorously shaken, incubated for 3 min at room temperature, subsequently centrifuged at 12,000 *g* at 4 °C for 15 min, and the upper (aqueous) phase containing the RNA was transferred to a fresh tube. 1 mL of ice-cold isopropanol was added, and the RNA was precipitated at − 20 °C for 1 h. After centrifugation at 12,000 g at 4 °C for 15 min, the RNA pellet was washed with 70% EtOH, air dried and resuspended in water.

For northern blot analyses, samples of 4 µg total RNA were mixed with sample buffer (40 mM 3-(N-morpholino)propanesulfonic acid (MOPS) pH 7.0, 50% formamide, 7% formaldehyde, 10 mM NaCl, 1 mM EDTA, 10% glycerol, 0.1% ethidium bromide, 0.02% bromophenol blue, 0.02% xylene cyanol), denatured at 75 °C for 15 min, shortly cooled on ice, then loaded on a denaturing 1% agarose gel containing 40 mM MOPS pH 7.0, 10 mM NaCl, 1 mM EDTA and 6% formaldehyde, and separated by gel electrophoresis for approximately 4 h at 80 V and 4 °C. The gels were subsequently blotted onto Hybond-N membranes (GE Healthcare) using capillary transfer with 10 × SSC buffer overnight. The blotted RNA was UV cross-linked to the membrane and stained with methylene blue. The 5′ UTR sequence of the actin gene amplified from pASH1 with the primer pair 5′-GCAAGGCGTACCGGAGAAG-3′/5′-CATGTTGCCTGCACTCCTCG-3′ was used as probe and labeled with [α-^32^P]dCTP by random priming (Megaprime™ DNA labeling system; GE Healthcare). Hybridizations were performed at 65 °C using standard protocols.

### Protein extraction and immunoblot analyses

Total soluble protein was isolated from cultured algae as described previously (Li and Bock 2018) with minor modifications. Disruption of cells was performed with a Sonifier®, W-250 D (G. Heinemann, Lorch, Germany) at 20% amplitude 3 times for 10 s on ice. Total cellular proteins were extracted from the cell lysate using a modified phenol/methanol method (Chatterjee et al. 2012). Briefly, cells were resuspended in extraction buffer (30% sucrose, 2% SDS, 10 mM Tris pH 8.0, 2% β-mercaptoethanol, 1 × cOmplete™ protease inhibitor cocktail, EDTA-free (Roche)), sonicated at 20% amplitude 3 times for 10 s on ice, and then mixed with an equal volume of phenol (pH 7.6). The sample was vortexed vigorously and centrifuged at 12,000 *g* at 4 °C for 10 min. The upper phenolic phase was collected, and proteins were precipitated with 4 volumes of ice-cold 0.1 M ammonium acetate in 100% methanol at − 20 °C overnight. After centrifugation at 12,000 *g* at 4 °C for 5 min, the protein pellet was washed twice with ice-cold methanol, and resuspended in protein buffer (50 mM Tris HCl pH 7.6, 100 mM NaCl, 10 mM KAc, 5 mM MgAc, 0.5% SDS, 1 × cOmplete™ protease inhibitor cocktail, EDTA-free (Roche)). Proteins were quantified using the BCA assay (Thermo Fisher Scientific).

For immunoblotting, samples were denatured at 95 °C for 5 min in 1 × SDS sample buffer (62.5 mM Tris–HCl pH 6.8, 10% glycerol, 2% SDS, 2% β-mercaptoethanol, 0.015% bromophenol blue), and electrophoretically separated in denaturing 12% SDS-PAA gels. Proteins were blotted onto nitrocellulose membranes (Amersham™Protran®, 0.45 µm pore size; GE Healthcare) using a semidry electroblotter (PeqLab, Germany) at 0.8 mA/cm^2^ for 75 min. Recombinant GFP (Thermo Fisher Scientific, Cat. No. 11814524001) was used as standard for semiquantitative assessment of protein accumulation. Protein detection was performed using a 1:1000 dilution of monoclonal anti-GFP primary antibody (Cat. No. 632381, Takara Bio, Saint-Germain-en-Laye, France) and a 1:25,000 dilution of anti-mouse HRP-conjugated secondary antibody (Agrisera, Vännäs, Sweden; Cat. No. AS111772). Chemiluminescence was detected with the ECL system (Thermo Fisher Scientific) and a G:BOX Chemi (Syngene).

### Protein precipitation from the culture medium

Collected medium was centrifuged at 12,000* g* at 4 °C for 10 min, and transferred to a fresh centrifuge tube. CTAB was added to a final concentration of 0.2% and the sample was vortexed rigorously. Precipitated polysaccharides were removed and 1 to 2 µg BSA per mL medium were added (as carrier) to the supernatant. Trichloroacetic acid (TCA) was added to a final concentration of 15%, and the sample was incubated on ice for 30 min to allow proteins to precipitate. After centrifugation at 20,000 *g* at 4 °C for 10 min, the pellet was washed twice with ice-cold methanol supplemented with 0.1 M NaAc. The protein pellet was air dried and resuspended in 1 × SDS sample buffer for gel electrophoresis.

### Bioinformatic analyses

RNAseq datasets (SRA Accession ERP111278) were analyzed to identify highly transcribed genes in *P.* *purpureum* using the Galaxy platform (Afgan et al. 2018). In this dataset, cells were grown in ASW medium at 25 °C in a 12 h light/12 h dark photoperiod at a light intensity of 100 µmol photons m^−2^ s^−1^. Samples were collected every two hours in triplicates, with the first sample collected one hour after the onset of the light. For the analysis of the expression data, samples taken after 5 h, 7 h and 9 h of light were pooled (to a total of 9 replicates). The updated genome sequence of strain CCMP1328 was used as reference genome for mapping (NCBI accession VRMN01; Lee et al. 2019).

Quality control of the FASTQ data was done with FastQC, followed by mapping of RNAseq reads with RNAStar using standard settings. The mapped reads were counted with FeatureCount, and normalized over all replicates to produce the final counts using DESeq2. Transcripts per million (TPM) values were calculated according to published methods (Wagner et al. 2012). As the goal of the analysis was to identify the most transcribed genes over all nine replicates (rather than the analysis of differential expression), genes showing a significant difference over the three time points were omitted. Genes with the top normalized counts were analyzed by BLAST (https://blast.ncbi.nlm.nih.gov/Blast.cgi) against the non-redundant protein sequence database and, if no hit was obtained, a search for conserved domains was performed.

## Results

### An improved transformation protocol for *Porphyridium purpureum*

In a previous study, we developed a transformation method for the red microalga *Porphyridium purpureum*. The method relies on biolistic DNA delivery of circular plasmid vectors that, rather than integrating into the nuclear genome, are maintained as autonomously replicating episomes in the nucleus (Li and Bock 2018). In subsequent sets of transformation experiments, we noticed that the efficiency of colony formation from single cells represented a bottleneck in *Porphyridium* transformation. To address this limitation, we tested different plating methods, and found that mixing of the algal cells with a starch suspension prior to plating on agar-solidified artificial sea water medium greatly increased the recovery of colonies from single cells (Supplementary Fig. 1a). Alternatively, embedding the cells in a thin film of low-density agar also substantially improved colony formation (Supplementary Fig. 1b).

In the previously established transformation protocol, cultured *Porphyridium* cells were plated on zeocin-containing medium, then bombarded with plasmid vector-coated gold particles, and regenerated into resistant colonies on the same agar plate. To reduce the cellular stress from immediate antibiotic exposure, we tested alternative protocols, in which cells are bombarded on medium without antibiotics, then washed off the plates, allowed to recover from particle bombardment in antibiotic-free liquid medium for different time periods, and subsequently plated on selective (zeocin-containing) medium. Transfer of colonies into liquid medium supplemented with zeocin was performed approximately 3 weeks after bombardment. The optimized procedure (Supplementary Fig. 2) increased the number of transgenic colonies approximately tenfold compared to the previous protocol. Due to the limited stability of the selection agent zeocin, the prolonged incubation time on plates compared to the previously published protocol (Li and Bock 2018) should not be extended beyond 3 weeks, although false positive colonies that start emerging after 3 weeks remain tiny and are yellowish, and thus can be easily distinguished from true transgenic colonies (that are large and red).

### Design of *YFP* gene variants for nuclear expression in *Porphyridium*

**Fig. 1:**
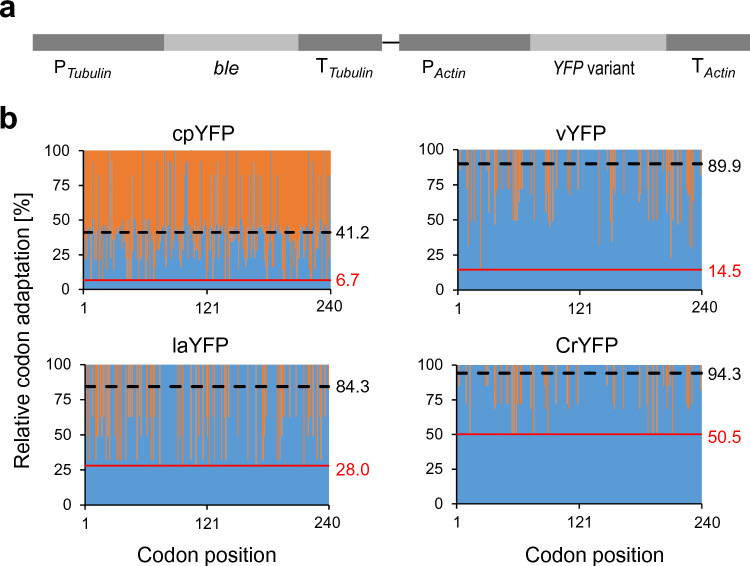
Physical map of the basic expression vector used for transformation of *Porphyridium purpureum*, and codon adaptation of the *YFP* gene variants tested for nuclear expression. **a** Physical map of the transformation vector used for expression of the *YFP* gene variants in *Porphyridium.* The zeocin resistance gene *ble* was placed under the control of the tubulin gene promoter and 5′ UTR (P_*Tubulin*_) and the tubulin gene terminator (T_*Tubulin*_). The different *YFP* gene variants were expressed under the control of the actin gene promoter and 5′ UTR (P_*Actin*_) and the actin gene terminator (T_*Actin*_). **b** *YFP* gene variants used in this study and their relative codon adaptation (RCA) compared to the codon usage in the nuclear genome of *Porphyridium* (see Supplementary Table 1). The RCA was plotted against the codon position, and blue bars indicate the relative adaptation of each codon (in %). The black number at the right indicates the average adaptation and the red number the minimal adaptation of each gene variant. The fully codon-optimized variant for *Porphyridium* (PpYFP) is not shown here, because, in this gene, all RCAs are at 100%

To assess the impact of GC content and codon usage on the efficiency of nuclear transgene expression in *Porphyridium*, we designed five versions of the gene encoding the yellow fluorescence protein (YFP). The five gene variants are identical with respect to the encoded amino acid sequence, but differ in their codon usage and GC content (Fig. 1). The codon usage of the variants was quantitatively assessed by the relative codon adaptation value (RCA; Fig. 1b), a reference set-based index that outperforms the previously used codon adaptation index (CAI) as reliable predictor of transgene expression efficiency (Fox and Erill 2010; Barahimipour et al. 2015).

The cpYFP variant is an AT-rich *YFP* gene version that was initially designed for expression in the chloroplast genome of the green alga *Chlamydomonas reinhardtii* (Barahimipour et al. 2015). Chloroplast genomes are highly AT-rich (Shimada and Sugiura 1991; Maul et al. 2002) and display a strong bias towards the use of synonymous codons having A or T in the third position. Since the *Porphyridium* nuclear genome is GC rich (with a GC content of 55.5%), the cpYFP variant has a very low average RCA of only 41.2% (Fig. 1b). The Venus variant of YFP (encoded by the *vYFP* gene; Fig. 1) was optimized as a reporter for mammalian cells (Nagai et al. 2002), and therefore, has a relatively high GC content (of 62%). Its overall RCA is also relatively high (89.9%), but it still contains a number of codons that are rarely used in *Porphyridium* (Fig. 1b).

The laYFP variant is a lowly codon-adapted gene variant that was designed to have a similarly high GC content as the *Porphyridium* nuclear genome, while being enriched for rarely used codons (Fig. 1b). This was done to be able to distinguish between the impact of GC content *versus* codon usage, and was accomplished largely by the introduction of synonymous G-to-C or C-to-G substitutions (Supplementary Table 1; Barahimipour et al. 2015).

The fourth *YFP* gene variant, CrYFP, is fully codon-optimized for the *Chlamydomonas* nuclear genome (Merchant et al. 2007). Although the *Chlamydomonas* and the *Porphyridium* nuclear genomes are both rich in GC and their codon usage is similar (average RCA: 94.3%), the CrYFP harbors several suboptimal codons, but lacks rare codons (minimal adaptation > 50%; Fig. 1b). Finally, a fifth gene variant, named PpYFP, was designed that is fully codon-optimized for *Porphyridium* and, consequently, has an RCA of 100%. To facilitate codon optimization, we assembled a codon usage table for the *Porphyridium* nuclear genome (Supplementary Table 1) based on a set of highly expressed intron-free genes (see Materials and methods). The sequences of all *YFP* gene variants can be found in Supplementary Table 4.

The five *YFP* gene versions were inserted into the identical expression cassette and vector backbone for *Porphyridium* nuclear transformation (Li and Bock 2018, 2019; Fig. 1a). The resulting constructs were bombarded into algal cells, and transgenic clones were selected for zeocin resistance conferred by the *ble* resistance gene (Li and Bock 2018).

### YFP accumulation in transgenic *Porphyridium* strains

**Fig. 2:**
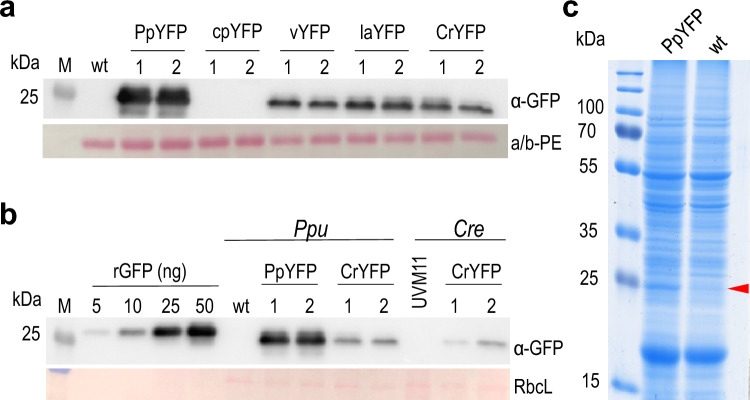
Protein expression from the different *YFP* variants and comparison to the green microalga *Chlamydomonas reinhardtii.* **a** Immunoblot analysis to compare YFP accumulation in transgenic algal strains. Samples of 5 µg total soluble protein (TSP) extracted from transformed *Porphyridium* clones expressing the different gene variants (cf. Fig. 1) were electrophoretically separated, blotted and immunodecorated with anti-GFP antibodies. Note that the perfectly codon-optimized gene version (PpYFP) results in the highest protein accumulation levels, whereas the poorly adapted AT-rich variant (cpYFP) shows no detectable expression. Ponceau staining of the α/β-phycoerythrin band (a/b-PE) on the blotted membrane served as loading control. M, molecular weight marker; wt, wild-type *P. purpureum*. **b** Semi-quantitative immunoblot analysis to determine YFP expression levels from fully codon-optimized gene variants in *P. purpureum* (PpYFP) and *C.* *reinhardtii (*CrYFP*).* 1 µg TSP was loaded for *P.* *purpureum* and *C. reinhardtii* and compared to a dilution series of recombinant GFP (Roche). YFP accumulates in *Porphyridium* to approximately 5% of TSP from the gene fully codon optimized for *P.* *purpureum* (PpYFP) lines, and to approximately 1.2% from the gene codon optimized for *C. reinhardtii* (CrYFP) lines. *Chlamydomonas* expression strain UVM11 (Neupert et al. 2009, 2020) accumulates the CrYFP to approximately 0.8% of TSP (Barahimipour et al. 2015)*.* Ponceau staining of the region of the blotted membrane containing the large subunit of Rubisco (RbcL) served as loading control. **c** Visualization of PpYFP accumulation in *P.* *purpureum* by Coomassie staining of 10 µg electrophoretically separated total soluble protein. The YFP can be seen as a distinct band at 25 kDa in the PpYFP transgenic strain

For each construct, several transgenic algal strains were isolated and their YFP accumulation levels were determined by immunoblotting (Fig. 2a, b). Due to the episomal maintenance of the transgenic constructs, expression levels are unaffected by position effects, and the only theoretical source of variable expression should be varying copy numbers of the plasmid, which however, are relatively constant (at approximately 20 copies per cell; Li and Bock 2018). Nonetheless, some heterogeneity in expression was observed in the primary transformants, and shown to be due to contamination with wild-type cells that had escaped antibiotic selection. This was remedied by single-colony purification (see below), and homogeneous cultures of transgenic cells were used for all subsequent analyses. As expected, the purified strains showed only little variability in expression levels, when independent transgenic clones produced with the same transformation vector were compared (Fig. 2a, b).

In the cpYFP transformants expressing the AT-rich gene version, YFP accumulation levels were below the detection limit (Fig. 2a), indicating that AT-rich genes with low RCA cannot be efficiently expressed in *Porphyridium*. All of the other four gene variants displayed readily detectable YFP accumulation (Fig. 2a). The fully codon-optimized gene version expressed in the PpYFP strains gave by far the highest YFP accumulation levels, suggesting that, beyond GC richness, optimal codon usage substantially enhances transgene expression (Fig. 2a). Analysis of cells by confocal laser-scanning microscopy revealed bright yellow YFP fluorescence, as expected (Supplementary Fig. 3).

Quantification of YFP accumulation in the transgenic strains revealed very high expression levels of approximately 5% of TSP from the fully codon-optimized gene variant (PpYFP), and approximately 1.2% from the gene codon optimized for *C. reinhardtii* (CrYFP). For comparison, we also analyzed transgenic *C. reinhardtii* strains that harbor the codon-optimized CrYFP in the nuclear genome of the expression strain UVM11 (Neupert et al. 2009, 2012; Barahimipour et al. 2015) under the control of the strong *PSAD* promoter. While this resulted in detectable YFP expression (as reported previously; Barahimipour et al. 2015), the YFP accumulation levels were substantially lower than in *Porphyridium* and reached only approximately 0.8% of TSP (Fig. 2b)*.*

Given the unprecedented accumulation of the PpYFP to 5% of TSP, we attempted detection of the recombinant protein by Coomassie staining of gel electrophoretically separated total cellular protein extracts. Indeed, these experiments revealed the presence of a prominent band of the expected size of YFP (Fig. 2c), thus confirming the very high expression of the fully codon-optimized transgene.

### Relationship between *YFP* mRNA and protein accumulation

**Fig. 3:**
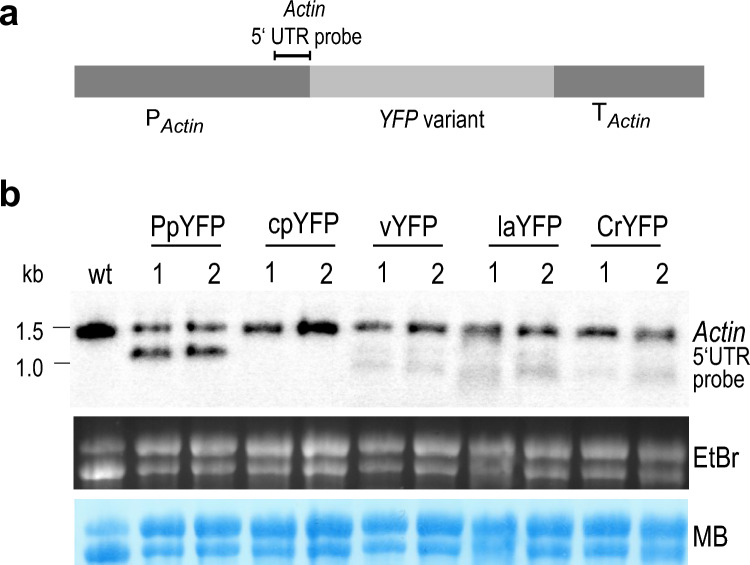
Northern blot analysis of *YFP* mRNA accumulation from the various gene variants expressed in *P.* *purpureum*. **a** Position of the actin 5′ UTR probe designed to (i) simultaneously detect all five *YFP* variants at equal sensitivity, and (ii) detect the endogenous actin mRNA as internal control. **b** RNA gel blot analysis of *YFP* transcript accumulation. Samples of 4 µg of total cellular RNA extracted from the same transgenic strains analyzed by western blotting (Fig. 2) were electrophoretically separated in a 1% denaturing agarose gel, blotted and hybridized to the radioactively labeled actin 5′ UTR probe indicated in panel (**a**). While the fully codon optimized *YFP* variant shows mRNA accumulation (~ 1.1 kb signal) to levels comparable to those of the endogenous actin transcript (~ 1.5 kb signal present also in the wild type; wt), all other variants show low transcript accumulation and evidence of partial mRNA degradation. Equal loading was verified by UV light detection of rRNA bands using ethidium bromide (EtBr), and blotting efficacy was checked by membrane staining with methylene blue (MB)

The observed differences in protein accumulation conferred by the different YFP variants prompted us to examine mRNA accumulation levels in the different transgenic red algal strains by RNA gel blot analyses (Fig. 3). To be able to accurately quantify mRNA levels by comparison to an internal standard, a probe was designed that simultaneously recognizes the transgenic mRNA and the endogenous actin mRNA (Fig. 3a). Due to the size difference of the two transcripts (with the actin mRNA being approximately 400 nt longer than the *YFP* mRNA; Fig. 3b), it is possible to clearly distinguish the two hybridization signals and normalize *YFP* transcript abundance to the endogenous actin mRNA signal.

The northern blot experiments revealed a clear positive correlation between protein accumulation and mRNA abundance (Fig. 3b). The transgenic algal clones generated with the cpYFP construct (that had shown undetectably low protein abundance; Fig. 2a) also displayed undetectably low *YFP* mRNA levels (Fig. 3b). vYFP, laYFP and CrYFP transformants showed weak hybridization signals for the *YFP* transcripts that were considerably weaker than the signal for the internal standard (i.e., the actin mRNA). Transgenic strains generated with the fully codon-optimized *YFP* gene (PpYFP) accumulated by far the highest *YFP* mRNA levels. Given that all transgenes are expressed from identical cassettes that reside in identical transformation vectors, the observed correlation between protein abundance and mRNA accumulation suggests that translating ribosomes stabilize the *YFP* transcripts. Consistent with this assumption, vYFP, laYFP and CrYFP transformants showed additional hybridization signals that are smaller than the full-length *YFP* mRNA and likely represent degradation intermediates.

Like the actin gene, the *YFP* transgene is also driven by the actin promoter. Interestingly, the intensity of the actin mRNA hybridization signal and that of the *YFP* mRNA were comparably strong in the PpYFP lines, indicating that, if codon usage is optimal, promoter strength is the main determinant of mRNA accumulation. In addition, this finding may indicate that there are no epigenetic factors influencing transcription from the two promoter copies.

### Testing a set of promoters from highly transcribed genes in *Porphyridium*

**Fig. 4:**
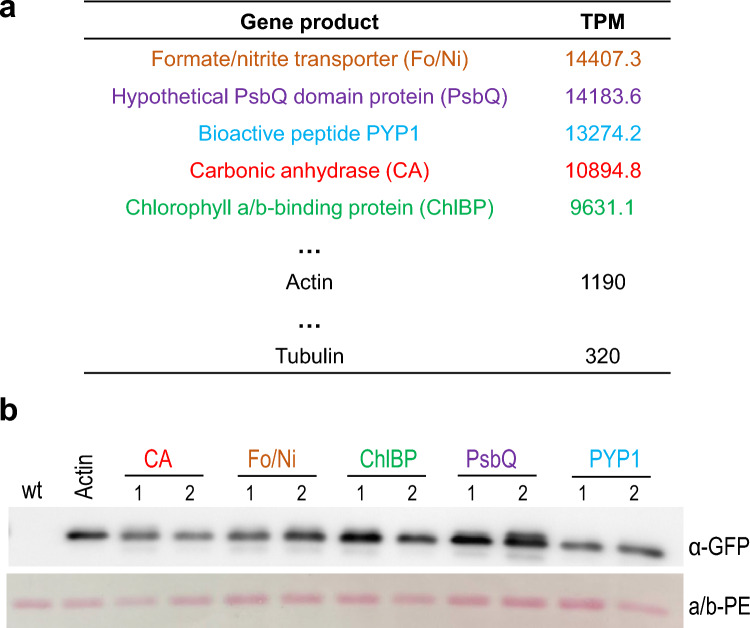
Analysis of the expression capacity of the five endogenous promoters that confer the highest mRNA accumulation levels in *P.* *purpureum*. **a** List of highly expressed genes in *Porphyridium* based on the analysis of RNAseq datasets (SRA Accession ERP111278; Supplementary Table 2). The top five transcripts show 10 to 40 times higher transcript per million (TPM) counts than the actin and tubulin mRNAs, whose promoters and 5′ UTRs have been used in previous experiments (Figs. 1 and 3). To assess their strength in a heterologous context, the five promoters were cloned in transformation vectors upstream of PpYFP (Fig. 1a). **b** Accumulation of YFP under the control of the five strong endogenous promoters in comparison to the actin promoter. Samples of 2 µg total protein were separated by gel electrophoresis, and YFP accumulation was examined using the identical *YFP* transgene controlled by the actin promoter as reference. Small increases in YFP accumulation are observed in the transgenic strains expression YFP under the control of the chlorophyll a/b-binding protein promoter (ChlBP) or the hypothetical PsbQ domain protein promoter (PsbQ). Ponceau staining of the α/β-phycoerythrin band (a/b-PE) on the blotted membrane served as loading control

The above-described analysis of the influence of codon usage and GC content on transgene expression was conducted with the established actin gene promoter, while the selectable marker gene *ble* was driven by the tubulin gene promoter. The two promoters were expected to be constitutively expressed to reasonable levels, and suitable to drive transgene expression in *Porphyridium* (Li and Bock 2018). Since promoter strength is an important determinant of transgene expression, we wanted to explore the possibility to further boost foreign protein accumulation by maximizing transgene transcription rates. To this end, we analyzed available RNAseq datasets of *Porphyridium* (SRA Accession ERP111278) to identify the most highly expressed mRNAs (Supplementary Table 2). The top genes of the resulting list have a more than tenfold higher mRNA accumulation level than actin and tubulin. The top five genes (excluding FVE85_6371 due to problems with PCR amplification and cloning) were then chosen (Fig. 4a), and fragments upstream of the translation initiation codon were cloned as putative promoters into our YFP expression vector to replace the actin promoter. Depending on the size of the intergenic spacer upstream of the gene of interest, fragments between 1500 and 500 bp were taken (1500 bp for CA, 500 bp for PsbQ, and 1000 bp for the other three genes). Transgenic algal clones were produced and YFP accumulation levels were analyzed by immunoblotting.

The results revealed that protein accumulation levels did not correlate with mRNA abundance of the corresponding endogenous genes, suggesting that there is substantial regulation of gene expression at the post-transcriptional level, presumably at the level of mRNA stability. Overall, the differences in protein accumulation were substantially smaller than the differences in mRNA accumulation (Fig. 4a, b). Notably, the promoter of the most strongly expressed endogenous gene, a putative formate/nitrite transporter, did not confer increased YFP accumulation. By contrast, increased YFP accumulation was seen with the promoter of the second most abundant endogenous mRNA, encoding a hypothetical PsbQ domain protein, and the promoter of a chloroplast-targeted chlorophyll-binding protein (ChlBP; Fig. 4). Together, these results indicate that, while promoter choice clearly influences recombinant protein accumulation, there is limited potential to substantially boost expression levels beyond 5% of TSP, presumably due to post-transcriptional control of gene expression in *Porphyridium* and/or differences in chromatin organization between the nuclear chromosomes and the (bacterial-type) plasmids in the nucleus.

### Targeting to the secretory pathway leads to efficient secretion of YFP into the culture medium

**Fig. 5:**
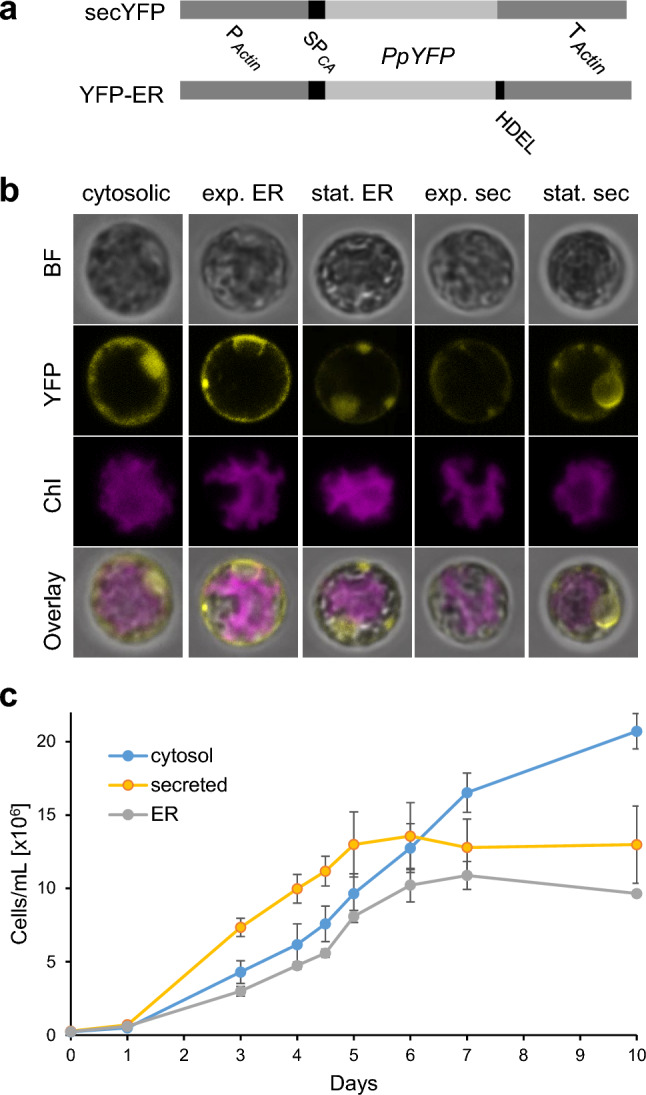
Targeting of YFP to the ER and the secretory pathway. **a** Map of the *YFP* expression cassettes in the transformation vectors used for targeting to the ER or the secretory pathway. The actin promoter, 5′ UTR and terminator, and the signal peptide sequence from the *CARBONIC ANHYDRASE 1* gene were used for both protein secretion and ER targeting. An additional HDEL motive was included at the C-terminal end of the ER construct to confer ER retention. **b** Confocal microscopy images of *Porphyridium* cells expressing cytosolic, ER-targeted or secreted (sec) YFP versions in the exponential (exp.) and stationary (stat.) growth phase. The bright field images (BF), the YFP fluorescence (YFP), the chlorophyll fluorescence (Chl) in the single star-shaped chloroplast and the overlay of the three images are shown. Note virtually complete absence of YFP fluorescence from cells expressing the secreted version in the exponential growth phase, but substantial YFP retention (presumably in the ER and/or Golgi apparatus) in the stationary phase. **c** Growth curves of *Porphyridium* cell cultures expressing cytosolic, secreted and ER-targeted YFP versions. Slightly lower cell numbers of the secYFP and YFP-ER strains in the stationary phase suggests a small growth penalty, presumably resulting from overloading the secretory pathway with large amounts of recombinant protein. Samples for microscopy were taken on days 3 and 10

Recombinant protein secretion from cultured cells or tissues into the medium represents a highly attractive strategy. By obviating the need to extract proteins from cells and purify the recombinant protein out of a highly complex mix of cellular proteins, secretion greatly simplifies product purification and reduces the costs of downstream processing (Borisjuk et al. 1999; Hellwig et al. 2004; Ramos-Martinez et al. 2017). To investigate whether *Porphyridium* can be engineered to efficiently secrete recombinant proteins into the culture medium, we constructed a YFP version destined for the secretory pathway (secYFP; Fig. 5a), by fusing the 21 amino acid signal peptide from the extracellular carbonic anhydrase, an abundant secreted protein (Fig. 4a; Supplementary Table 2), to the YFP N-terminus. As a control, we produced a YFP version that, in addition to the signal peptide for secretion, harbored a C-terminal ER retention signal (YFP-ER; Fig. 5a), and therefore, is expected to accumulate intracellularly in the ER.

The two expression constructs were introduced into algal cells by biolistic transformation, and the YFP accumulation patterns were analyzed by confocal laser-scanning microscopy (Fig. 5b). The YFP-ER protein accumulated in distinct structures within the cytosol, presumably representing the endoplasmic reticulum. By contrast, the secYFP was hardly detectable in exponentially growing cells, possibly suggesting efficient secretion out of the algal cells. However, in the stationary phase, significant intracellular retention of the YFP fluorescence signal was seen, indicating that, at this stage, a substantial proportion of the synthesized YFP remains trapped in the ER and/or Golgi apparatus. This observation may suggest that, in the stationary phase, protein secretion becomes less efficient.

The growth rate of the algal cultures was not appreciably affected by targeting of the foreign protein to the ER or the secretory pathway (Fig. 5c). However, the cell density reached in the stationary phase was slightly lower than in the control cultures accumulating YFP in the cytosol (Fig. 5c). This may be indicative of cellular stress induced by overloading the ER with high levels of constitutively synthesized YFP in the YFP-ER and secYFP strains.

**Fig. 6:**
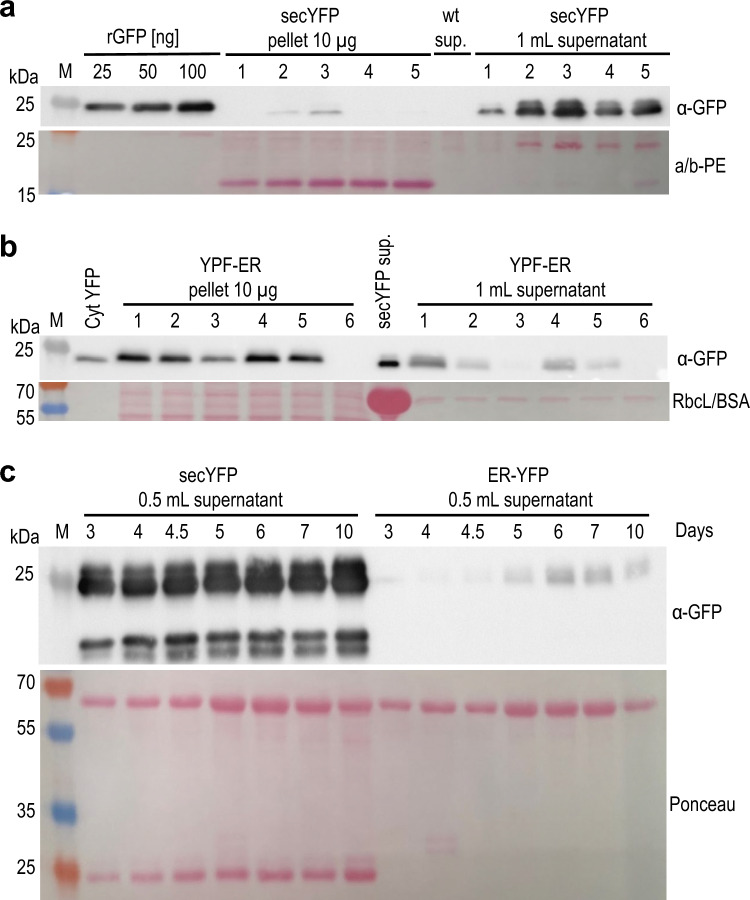
Secretion and ER retention of YFP in *Porphyridium.* **a** Western blot analysis to detect YFP in *Porphyridium* strains engineered to secrete the protein into the culture medium (cf. Fig. 5). Samples of 10 µg total protein extracted from cells washed and pelleted were electrophoretically separated by SDS-PAGE, blotted and immunodecorated with anti-GFP antibodies (α-GFP). Proteins precipitated with TCA from aliquots of 1 mL culture medium (supernatant; sup.) were loaded to assess YFP secretion. Five independently generated secYFP transformants were analyzed. For semiquantitative analysis of YFP accumulation, a dilution series of recombinant GFP was loaded. While high levels of YFP accumulate in the medium, only very low amounts of protein are detectable in the washed cell pellet. Clone secYFP-3 was used in all further experiments. Ponceau staining of the α/β-phycoerythrin band (a/b-PE) on the blotted membrane served as loading control. M: molecular weight marker; wt: wild type. **b** Western blot detection of YFP in transgenic *Porphyridium* cultures targeting the protein to the ER. Samples of 10 µg total protein extracted from pelleted cells was analyzed and compared to 1 mL of the culture medium. As positive controls, 2 µg total protein extracted from an algal strain expressing the cytosolic PpYFP (Cyt YFP) positive control, and the equivalent supernatant (60 µL) of a secYFP strain were included. As expected, the YFP is mainly present in the cell pellet, and only small amounts are found in the medium. YFP-ER strain 4 was used in all subsequent experiments. Ponceau staining of the region of the blotted membrane containing the large subunit of Rubisco (RbcL) served as loading control. BSA was used as precipitation carrier for the secYFP control and gives rise to a strong band on the membrane. **c** Accumulation of YFP in the culture medium of strains expressing secYFP or YFP-ER over 10 days. Samples were collected during the growth experiment depicted in Fig. 5c. secYFP accumulation in the medium increased with cultivation time. The double band of YFP (migrating at ~ 25 kDa) is likely explained by incomplete cleavage of the N-terminal secretion signal. The two bands of lower molecular weight represent putative degradation intermediates. Ponceau staining of the blotted membrane was conducted as control for equal loading, and is shown below the blot. Note that YFP accumulates as the only prominent protein present in the culture medium. The other strong protein band (at ~ 66 kDa) is BSA that was added as precipitation carrier

Next, we tested for the presence of YFP in the culture medium of the secYFP-expressing transgenic algae. Indeed, protein precipitation from the medium detected high levels of YFP, whereas the cell pellet contained only very low amounts of the protein (Fig. 6a). By contrast, the YFP-ER is mainly found in the cell pellet, as expected, and only small amounts are present in the culture medium (Fig. 6b). These small amounts of YFP in the medium could come from leaky secretion or, alternatively, from a small fraction of lysed cells present in the culture.

Analysis of YFP accumulation in the culture medium over time confirmed the efficient secretion of secYFP and the low-level presence of the YFP-ER (Fig. 6c). Only a faint YFP signal can be detected in the supernatant of the ER-targeted YFP lines, consistent with (i) the ER retention signal efficiently keeping the YFP within the cell, and (ii) the rate of cell rupture being very low (and not contributing substantially to the strong YFP signal in the supernatant of the secYFP strains). secYFP accumulation in the medium increased with cultivation time. Ponceau staining of the secreted proteins revealed that YFP is highly abundant in the secreted proteome, and accumulates as the only secreted protein that appears as prominent band in the gel (Fig. 6c). These results indicate a low complexity of the secreted proteome of *Porphyridium*, and suggest a straightforward purification of secreted recombinant proteins from the culture medium. Immunoblot analysis also detected some (less abundant) putative degradation products of YFP (Fig. 6c), possibly indicating that *Porphyridium* also secretes proteases into the medium.

**Fig. 7:**
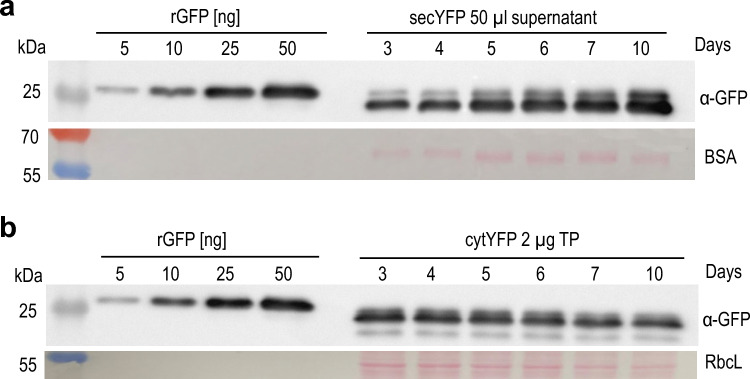
Quantification of YFP accumulation in transgenic *Porphyridium* strains expressing the secreted or the cytosolic version of YFP. **a** Time course of secYFP accumulation in the culture medium of *Porphyridium* over 10 days. The initial cell density was 2.73 × 10^5^ cells/mL, and the samples were collected during the growth experiment shown in Fig. 5c. Aliquots of 50 µL medium were loaded and compared to a dilution series of a recombinant GFP standard. At day 10, YFP accumulation in the medium reached approximately 1.6 mg/L. BSA was added to the collected supernatant as precipitation carrier, and the region containing the BSA band is shown in the Ponceau stain below the blot. Relevant bands of the molecular weight marker are labeled (with the sizes in kDa). **b** Time course of cytYFP accumulation. Samples of 2 µg total protein were loaded and compared to the GFP standard. YFP accumulation in the cytosol remains largely constant during cultivation, with the expression level being approximately 2% of the total protein. Ponceau staining of the region of the blotted membrane containing the large subunit of Rubisco (RbcL) served as loading control. Note that the total soluble protein accounts for approximately half of the total cellular protein in *Porphyridium*

Quantification of secYFP in the culture medium confirmed the increase in protein accumulation with time (Fig. 7a). By contrast, cytosolic accumulation of YFP remained largely stable during the cultivation period, and accumulated to approximately 2% of the total protein. Comparison of the quantification of YFP accumulation relative to total cellular protein versus total soluble protein (Figs. 2b and 7b) suggests that the total soluble protein accounts for approximately half of the total cellular protein in *Porphyridium*.

YFP accumulation in the medium reached very high values of approximately 1.6 mg/L on day 10, suggesting protein secretion and harvest from the medium as an efficient strategy for recombinant protein expression in *Porphyridium*. When comparing the intracellular YFP accumulation of the cytosolic YFP (Fig. 2b) and the secYFP (Fig. 6a) with the GFP standard, it becomes obvious that nearly all YFP is effectively secreted out of the cell in the secYFP strains. For comparison, we calculated that a *Porphyridium* cell contains approximately 23.4 ± 2.2 pg protein (see Supplementary Table 4). Taken the cell number (as determined in Fig. 5c) on day 10 into account, the total intracellular protein content of a 1 mL *Porphyridium* culture amounts to 302.43 µg protein. This value corresponds to 1.6 µg secreted YFP per mL supernatant.

The occurrence of YFP as a double band in the supernatant of the secYFP lines (Fig. 6c) suggests incomplete cleavage of the signal peptide (SP) from the reporter protein. Usually, the SP is directly cleaved by the signal peptidase upon co-translational translocation into the ER. This cleavage can be less efficient, when the SP is placed in a non-native context (e.g., fused to a recombinant protein). This was observed previously, for example, with the SP of the BIP1 protein fused to a GFP reporter to facilitate ER targeting in *Chlamydomonas reinhardtii*, which also resulted in occurrence of a double band (Niemeyer et al. 2021).

### Stability of transgene expression

**Fig. 8:**
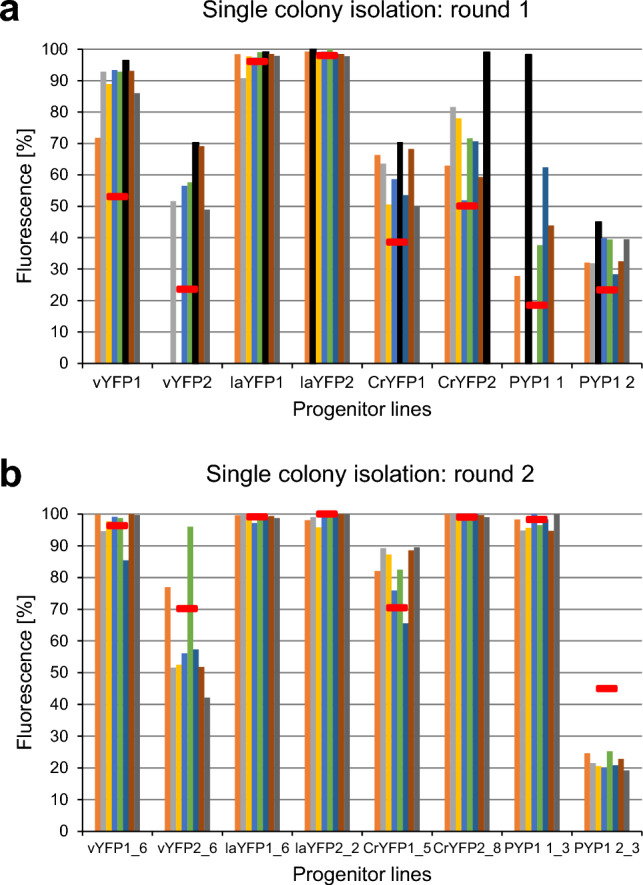
Purification of transgenic strains by single colony isolation. **a** First round of purification. Cells of the progenitor lines (with transgenic clone numbers indicated on the x-axis) growing in liquid culture and showing the proportions of fluorescing cells indicated by the red horizontal bars were plated on lsASW agar plates using the starch method to obtain single colonies (see “Materials and methods” section and Supplementary Figs. 1 and 2). Eight colonies per strain were randomly picked, and the ratio of fluorescent to non-fluorescent cells in liquid culture was determined by microscopy. After the first round of purification, all ratios were higher than those of the progenitor cultures (and reached 100% in several cultures). **b** Second round of purification. The subcultures indicated by the black bars in (a) were picked, and used for another round of single-colony purification. A large number of the subcultures is now homogeneously fluorescent

We have previously shown that transgene expression in *Porphyridium* remains stable over long periods of time (Li and Bock 2018). These previous findings were confirmed in our present study by showing unaltered transgene expression levels after 11 months of subcultivation (equivalent to 22 cultivation rounds, i.e., cell culture passages; Supplementary Fig. 4). However, in the course of the work described here, we noticed that many of the liquid cultures inoculated with primary transgenic algal colonies were not homogeneously fluorescent (Fig. 8). We tentatively attributed this to residual contamination with untransformed cells that may have been able to survive and grow in the shade of the zeocin-resistant transformed cells. Fluorescence-activated cell sorting (FACS) did not result in efficient purification of fluorescing cells, most likely due to cell aggregation caused by the sticky cell wall polysaccharides of *Porphyridium*. We, therefore, attempted to purify the transgenic cells to homogeneity by passing them through additional rounds of colony formation on plates followed by liquid culture and assessment of fluorescent versus non-fluorescent cells (Fig. 8).

Indeed, after two additional purification rounds, many of the cultures were homogeneously fluorescent (Fig. 8), and fluorescence remained stable and uniform during subsequent cultivation in liquid medium or on agar-solidified medium. All strains used in our analyses of codon usage and promoter strength (Figs. 2, 3, 4) were homogenously fluorescent, while the variation seen in the different YFP-ER lines and secYFP lines (Fig. 6) might be due to residual contamination with wild-type cells (in the strains showing lower expression levels).

It is noteworthy in this context that the selection agent zeocin is inhibited by high ionic strength (according to the Invitrogen zeocin data sheet), and the lsASW medium used for cultivation of *Porphyridium* contains more than 5 g salt/L. Thus, long incubation times upon primary selection of transgenic algal clones, together with the high ionic strength of the medium, may lower the effective zeocin concentration and allow wild-type cells to grow in the shade of transformed cells. The inherent stickiness of *Porphyridium* cells may further exacerbate this effect.

In view of these findings, we strongly recommend to pass all primary transformed algal clones through two or three additional rounds of selection and single colony isolation to obtain homogeneous cultures of transgenic algae.

## Discussion

In this work, we have investigated parameters that affect nuclear transgene expression in the red alga *Porphyridium purpureum*. We identified promoter choice and codon usage as important determinants of expression efficiency, and developed new vectors that allow recombinant protein secretion into the culture medium. Our comparative analysis of five different *YFP* gene versions that varied in GC content and codon usage confirmed that (i) AT-rich genes are difficult to express in GC-rich nuclear genomes, and (ii) similar to green algae (Barahimipour et al. 2015), codon usage is an important determinant of transgene expression efficiency, largely independent of the GC content of the coding region.

Our comparative analyses of YFP protein accumulation and *YFP* transcript accumulation in the transgenic algal strains expressing the different gene variants revealed a strong positive correlation between translational efficiency (determined by codon usage) and mRNA stability (Figs. 2 and 3). This finding suggests that high translation rates enhance mRNA stability, presumably by translating ribosomes shielding the mRNA from endoribonucleolytic attack. This conclusion gained further support from the detection of partially degraded transcripts in the strains expressing non-codon optimized YFP variants (Fig. 3b). Overall, our findings are in agreement with studies in yeast and *Chlamydomonas* that also reported a strong influence of codon usage (and, presumably, translation rates) on mRNA stability (Barahimipour et al. 2015; Presnyak et al. 2015).

Quantitation of YFP accumulation in the transgenic red algal strains expressing the fully codon-optimized gene variant revealed that YFP accumulated to approximately 5% of the total soluble protein. To our knowledge, this is one of the highest expression levels of a recombinant protein in algae reported to date. These high expression levels will expand the range of reporter protein-based cell biological methods that are usable in red algae, and will also stimulate new applications in algal biotechnology and synthetic biology.

Given the high costs associated with recombinant protein purification from cells and tissues, protein secretions into the culture medium represents an attractive option to minimize product losses and reduce the costs of downstream processing in molecular farming (Borisjuk et al. 1999; Hellwig et al. 2004; Ramos-Martinez et al. 2017). The efficient targeting of YFP to the secretory pathway in *Porphyridium* (Figs. 5, 6, 7) and the low complexity of the contaminating endogenous algal proteins present in the supernatant after pelleting of the cells (Fig. 6c) suggest protein secretion as a promising strategy for cost-effective recombinant protein production in red microalgae. While protein secretion was highly efficient in the exponential growth phase, it was less efficient in the stationary phase as evidenced by a significant fraction of the protein accumulating intracellularly (Fig. 5b). Whether this observation is explained by secretion becoming less efficient in the stationary phase, or alternatively, is linked to the formation of the thick polysaccharide cell wall characteristic of many unicellular red algae in the stationary phase, remains to be investigated.

Compared to the green alga *Chlamydomonas*, the red alga *Porphyridium* offers several potential advantages that may make it a superior expression host for recombinant protein production. First, as shown in this study, much higher expression levels are attainable in *Porphyridium*, when the same transgenes are expressed in the two algal species. Remarkably, even a gene variant that is fully codon-optimized for *Chlamydomonas* gives higher expression levels in transgenic *Porphyridium* strains than in transgenic *Chlamydomonas* strains (Fig. 2b). This may, at least in part, be due to the high copy number of the episomally maintained transgenes in the red alga. Second, the lack of transgene integration into nuclear chromosomes makes transgenes in *Porphyridium* insensitive to position effects, and potentially less prone to epigenetic transgene inactivation. Consistent with this assumption, high level transgene expression shows little variation between transgenic clones (Figs. 2a, b, 6). Also, transgene expression has been shown to persist in *Porphyridium* for more than half a year without any noticeable decline in recombinant protein accumulation levels (Li and Bock 2018). Whether or not *Porphyridium* lacks the epigenetic transgene inactivation mechanism recently identified in *Chlamydomonas* (Neupert et al. 2020) remains to be determined. Finally, *Porphyridium* has an extremely intron-poor genome (Bhattacharya et al. 2013), whereas the genome of *Chlamydomonas* is very rich in introns (and has a median intron number of 7.81 per gene; Craig et al. 2021). Consequently, intron inclusion into the coding regions of transgenes in *Chlamydomonas* is currently required to achieve expression especially of longer transgenes (Mussgnug 2015; Baier et al. 2018). By contrast, the *Porphyridium* genome contains only 235 spliceosomal introns, and many large genes are completely intron-free (Bhattacharya et al. 2013). Thus, *Porphyridium* is unlikely to require introns for efficient expression of nuclear (trans)genes. Indeed, preliminary experiments involving the expression of transgenes with substantially longer coding regions than *YFP* indicate that transgene expression efficiency is not negatively correlated with gene length (Hammel et al., manuscript in preparation).

The molecular mechanisms of plasmid maintenance in *Porphyridium* remains to be elucidated. The plasmids used in this work have a ColE1 origin of replication. In bacteria, replication of this plasmid family relies on synthesis of RNA II (by the RNA polymerase) that acts as pre-primer. Cleavage of RNA II by RNase H then facilitates extension of the leading strand by DNA polymerase I. Subsequently, the replication fork is assembled by the bacterial proteins PriA, PriB and DnaT, allowing the replicative DNA polymerase III to replicate the plasmid (Camps 2010). Interestingly, four DNA polymerase I homologs that are predicted to be targeted neither to the chloroplast nor to the mitochondria (FVE85_7267, FVE85_6497, FVE85_1954 and FVE85_1955) were found in the nuclear genome of *Porphyridium*. These DNA polymerase I homologs could potentially be involved in plasmid maintenance in the algal nucleus. It is also conceivable that eukaryotic replication factors present in the nucleus (including RNase H and eukaryotic DNA polymerases) are recruited to support plasmid replication in *Porphyridium*.

In sum, our work reported here (i) demonstrates the high potential of the red alga *Porphyridium purpureum* for recombinant protein production, (ii) identified codon usage as an important determinant of expression efficiency at both the protein and mRNA levels, and (iii) showed that foreign proteins can be efficiently targeted to the secretory pathway of the alga, leading to product secretion into the culture medium. Together, these findings provide new opportunities for algal biotechnology and synthetic biology, and suggest red microalgae as a highly competitive production system for molecular farming.

### Supplementary Information

Below is the link to the electronic supplementary material.

Supplementary file1 (DOC 2077 kb)

## Data Availability

The authors confirm that the data supporting the findings of this study are available within the article and its supplementary materials.
