# Supplementary material for: Optimized transgene expression in the red alga Porphyridium purpureum and efficient recombinant protein secretion into the culture medium
Source: Plant Mol Biol. 2024 Feb 14;114(1):18. doi: 10.1007/s11103-024-01415-2 (PMC10866757; doi:10.1007/s11103-024-01415-2)
Supplement: Supplementary file 1 — Supplementary file1 (DOC 2077 kb) [file 11103_2024_1415_MOESM1_ESM.doc]

**Supplementary Information**

**Supplementary Table 1.** Codon usage table of *Porphyridium purpureum.*

| **AAcid** | **Codon** | **Count** | **/1000** | **Usage** | **AAcid** | **Codon** | **Count** | **/1000** | **Usage** |
| --- | --- | --- | --- | --- | --- | --- | --- | --- | --- |
| Ala | GCG | 1314 | 48.59 | 0.43 | Leu | TTG | 334 | 12.35 | 0.16 |
| Ala | GCA | 446 | 16.49 | 0.15 | Leu | TTA | 13 | 0.48 | 0.01 |
| Ala | GCT | 390 | 14.42 | 0.13 | Leu | CTG | 865 | 31.99 | 0.42 |
| Ala | GCC | 922 | 34.1 | 0.3 | Leu | CTA | 29 | 1.07 | 0.01 |
| Arg | AGG | 154 | 5.7 | 0.11 | Leu | CTT | 250 | 9.25 | 0.12 |
| Arg | AGA | 67 | 2.48 | 0.05 | Leu | CTC | 561 | 20.75 | 0.27 |
| Arg | CGG | 102 | 3.77 | 0.07 | Lys | AAG | 1284 | 47.49 | 0.89 |
| Arg | CGA | 142 | 5.25 | 0.1 | Lys | AAA | 163 | 6.03 | 0.11 |
| Arg | CGT | 201 | 7.43 | 0.15 | Met | ATG | 891 | 32.95 | 1 |
| Arg | CGC | 707 | 26.15 | 0.51 | Phe | TTT | 473 | 17.49 | 0.4 |
| Asn | AAT | 198 | 7.32 | 0.2 | Phe | TTC | 709 | 26.22 | 0.6 |
| Asn | AAC | 777 | 28.74 | 0.8 | Pro | CCG | 667 | 24.67 | 0.51 |
| Asp | GAT | 412 | 15.24 | 0.31 | Pro | CCA | 279 | 10.32 | 0.21 |
| Asp | GAC | 931 | 34.43 | 0.69 | Pro | CCT | 119 | 4.4 | 0.09 |
| Cys | TGT | 70 | 2.59 | 0.16 | Pro | CCC | 247 | 9.13 | 0.19 |
| Cys | TGC | 380 | 14.05 | 0.84 | Ser | AGT | 118 | 4.36 | 0.06 |
| End | TGA | 19 | 0.7 | 0.21 | Ser | AGC | 470 | 17.38 | 0.24 |
| End | TAG | 30 | 1.11 | 0.33 | Ser | TCG | 710 | 26.26 | 0.36 |
| End | TAA | 43 | 1.59 | 0.47 | Ser | TCA | 145 | 5.36 | 0.07 |
| Gln | CAG | 803 | 29.7 | 0.84 | Ser | TCT | 210 | 7.77 | 0.11 |
| Gln | CAA | 156 | 5.77 | 0.16 | Ser | TCC | 343 | 12.68 | 0.17 |
| Glu | GAG | 1196 | 44.23 | 0.77 | Thr | ACG | 501 | 18.53 | 0.38 |
| Glu | GAA | 365 | 13.5 | 0.23 | Thr | ACA | 181 | 6.69 | 0.14 |
| Gly | GGG | 273 | 10.1 | 0.12 | Thr | ACT | 176 | 6.51 | 0.13 |
| Gly | GGA | 454 | 16.79 | 0.2 | Thr | ACC | 465 | 17.2 | 0.35 |
| Gly | GGT | 440 | 16.27 | 0.19 | Trp | TGG | 312 | 11.54 | 1 |
| Gly | GGC | 1120 | 41.42 | 0.49 | Tyr | TAT | 174 | 6.43 | 0.22 |
| His | CAT | 128 | 4.73 | 0.27 | Tyr | TAC | 601 | 22.23 | 0.78 |
| His | CAC | 344 | 12.72 | 0.73 | Val | GTG | 1145 | 42.34 | 0.55 |
| Ile | ATA | 16 | 0.59 | 0.01 | Val | GTA | 114 | 4.22 | 0.05 |
| Ile | ATT | 440 | 16.27 | 0.41 | Val | GTT | 259 | 9.58 | 0.12 |
| Ile | ATC | 612 | 22.63 | 0.57 | Val | GTC | 580 | 21.45 | 0.28 |
| AAcid: amino acid; Count: number of codons counted; /1000: occurrence per thousand codons; Usage: usage of this codon to encode the respective amino acid | | | | | | | | | |

**Supplementary Table 2.** Top 100 highly expressed endogenous genes in *Porphyridium purpureum*.

| **Locus tag** | **Gene product** | **TPM** |
| --- | --- | --- |
| FVE85_0107 | Formate/Nitrite transporter YrhG | 14407.33 |
| FVE85_3577 | hypothetical protein (PsbQ domain) | 14183.62 |
| FVE85_6364 | Protein PYP1 | 13274.17 |
| FVE85_3079 | Carbonic anhydrase | 10894.83 |
| FVE85_6371 | hypothetical protein FVE85_6371 | 10333.3 |
| FVE85_6435 | Chlorophyll a-b binding protein, chloroplastic | 9631.395 |
| FVE85_0111 | D-glycerate 3-kinase, chloroplastic | 8261.663 |
| FVE85_6090 | hypothetical protein FVE85_6090 | 7928.787 |
| FVE85_2042 | R-phycoerythrin gamma chain, chloroplastic | 7258.901 |
| FVE85_2041 | R-phycoerythrin gamma chain, chloroplastic | 6801.489 |
| FVE85_7927 | Chlorophyll a-b binding protein 1B-21, chloroplastic | 6426.702 |
| FVE85_5651 | Chlorophyll a-b binding protein of LHCII type III, chloroplastic | 6018.439 |
| FVE85_2505 | Fucoxanthin-chlorophyll a-c binding protein, chloroplastic | 5984.899 |
| FVE85_0338 | Chlorophyll a-b binding protein, chloroplastic | 5965.422 |
| FVE85_7382 | Chlorophyll a-b binding protein 1B-21, chloroplastic | 5828.118 |
| FVE85_3955 | Chlorophyll a-b binding protein 1B-21, chloroplastic | 5557.657 |
| FVE85_3924 | hypothetical protein FVE85_3924 | 5276.498 |
| FVE85_8023 | Phycobilisome 31.8 kDa linker polypeptide, phycoerythrin-associated, rod | 5070.958 |
| FVE85_2781 | Photosystem I subunit O | 4762.725 |
| FVE85_8322 | Photosystem II 12 kDa extrinsic protein, chloroplastic | 4672.513 |
| FVE85_6079 | hypothetical protein FVE85_6079 | 4565.703 |
| FVE85_6536 | hypothetical protein FVE85_6536 | 4481.354 |
| FVE85_1413 | hypothetical protein FVE85_1413 | 4469.18 |
| FVE85_9678 | Uncharacterized protein FVE85_9678 | 4457.982 |
| FVE85_7666 | 5-methyltetrahydropteroyltriglutamate--homocysteine methyltransferase | 4414.724 |
| FVE85_0106 | Carbonic anhydrase 2 | 4311.254 |
| FVE85_9240 | R-phycoerythrin gamma chain, chloroplastic | 3977.435 |
| FVE85_2942 | hypothetical protein FVE85_2942 | 3897.133 |
| *FVE85_8746* | *hypothetical protein FVE85_8746* | *3749.734* |
| FVE85_1414 | hypothetical protein FVE85_1414 | 3667.125 |
| FVE85_7594 | hypothetical protein FVE85_7594 | 3582.766 |
| FVE85_2309 | R-phycoerythrin gamma chain, chloroplastic | 3562.359 |
| FVE85_2293 | hypothetical protein FVE85_2293 | 3546.824 |
| FVE85_5340 | hypothetical protein FVE85_5340 | 3426.4 |
| FVE85_5355 | Oxygen-evolving enhancer protein 1, chloroplastic | 3295.069 |
| FVE85_8316 | Cytochrome b6-f complex iron-sulfur subunit 1, cyanelle | 3231.383 |
| FVE85_0135 | Cold shock domain-containing protein 4 | 3201.967 |
| *FVE85_4028* | *Cofilin* | *2917.666* |
| *FVE85_6962* | *hypothetical protein FVE85_6962* | *2899.28* |
| FVE85_6702 | Superoxide dismutase Mn, mitochondrial | 2844.892 |
| FVE85_6005 | hypothetical protein FVE85_6005 | 2765.933 |
| FVE85_7812 | Methanesulfonate monooxygenase ferredoxin subunit | 2664.067 |
| FVE85_8365 | Phycobilisome 31.8 kDa linker polypeptide, phycoerythrin-associated, rod | 2554.758 |
| FVE85_5468 | Ammonium transporter 1 member 2 | 2546.475 |
| FVE85_5305 | hypothetical protein FVE85_5305 | 2535.308 |
| FVE85_6170 | Zinc transporter ZupT | 2295.755 |
| FVE85_2375 | Ferrous iron permease EfeU | 2250.688 |
| FVE85_1592 | hypothetical protein FVE85_1592 | 2242.989 |
| FVE85_8359 | hypothetical protein FVE85_8359 | 2175.146 |
| FVE85_2640 | hypothetical protein FVE85_2640 | 2105.432 |
| *FVE85_2854* | *hypothetical protein FVE85_2854* | *2027.673* |
| FVE85_5140 | Linker RC5 | 2024.174 |
| FVE85_2471 | hypothetical protein FVE85_2471 | 2021.889 |
| FVE85_1715 | hypothetical protein FVE85_1715 | 1982.415 |
| *FVE85_6254* | *SNF1-related protein kinase regulatory subunit beta-2* | *1965.711* |
| FVE85_7087 | hypothetical protein FVE85_7087 | 1955.946 |
| FVE85_5222 | hypothetical protein FVE85_5222 | 1940.605 |
| FVE85_2240 | Aspartate--ammonia ligase | 1936.67 |
| FVE85_6016 | hypothetical protein FVE85_6016 | 1920.607 |
| *FVE85_2199* | *hypothetical protein FVE85_2199* | *1909.823* |
| FVE85_7221 | Phycobilisome 31.8 kDa linker polypeptide, phycoerythrin-associated, rod | 1865.696 |
| FVE85_2982 | hypothetical protein FVE85_2982 | 1863.984 |
| FVE85_0729 | Phycobilisome 27.9 kDa linker polypeptide, phycoerythrin-associated, rod | 1863.821 |
| FVE85_3889 | hypothetical protein FVE85_3889 | 1860.406 |
| FVE85_2465 | hypothetical protein FVE85_2465 | 1842.204 |
| FVE85_2818 | Phycobilisome 7.8 kDa linker polypeptide, allophycocyanin-associated, core | 1812.344 |
| FVE85_4479 | hypothetical protein FVE85_4479 | 1809.635 |
| FVE85_5869 | 6-phosphogluconolactonase | 1807.669 |
| FVE85_0134 | Cold shock domain-containing protein 4 | 1779.967 |
| FVE85_3229 | Elongation factor 1-alpha | 1731.754 |
| FVE85_2468 | hypothetical protein FVE85_2468 | 1728.796 |
| FVE85_1011 | Glycine-rich RNA-binding protein | 1723.927 |
| FVE85_5793 | hypothetical protein FVE85_5793 | 1718.107 |
| FVE85_9475 | hypothetical protein FVE85_9475 | 1701.586 |
| FVE85_6320 | Peroxisomal membrane protein 2 | 1688.202 |
| FVE85_4247 | Linker RC4 | 1675.684 |
| FVE85_8317 | Protein sym-1 | 1669.745 |
| FVE85_6037 | Zinc transporter ZIP1 | 1567.177 |
| FVE85_3578 | hypothetical protein FVE85_3578 | 1560.506 |
| FVE85_7258 | Linker RC6 | 1542.948 |
| FVE85_2314 | Phycobilisome 32.1 kDa linker polypeptide, phycocyanin-associated, rod | 1540.393 |
| FVE85_8246 | hypothetical protein FVE85_8246 | 1517.096 |
| FVE85_1265 | Fructose-bisphosphate aldolase 1, chloroplastic | 1513.443 |
| FVE85_1484 | hypothetical protein FVE85_1484 | 1507.37 |
| FVE85_8240 | Axial regulator YABBY 5 | 1440.153 |
| FVE85_6163 | hypothetical protein FVE85_6163 | 1423.781 |
| FVE85_3879 | Phycobilisome 31.8 kDa linker polypeptide, phycoerythrin-associated, rod | 1415.86 |
| FVE85_4096 | 14-3-3 protein epsilon | 1406.856 |
| FVE85_2264 | Bifunctional enolase 2/transcriptional activator | 1406.089 |
| FVE85_1945 | Ferredoxin--NADP reductase, cyanelle | 1400.638 |
| FVE85_8260 | Peroxiredoxin-6 | 1394.547 |
| FVE85_0385 | Carbonic anhydrase 2 | 1393.334 |
| FVE85_7126 | hypothetical protein FVE85_7126 | 1389.001 |
| *FVE85_7722* | *Calvin cycle protein CP12, chloroplastic* | *1383.908* |
| FVE85_7633 | hypothetical protein FVE85_7633 | 1381.935 |
| FVE85_0816 | Phycobilisome 27.9 kDa linker polypeptide, phycoerythrin-associated, rod | 1369.163 |
| FVE85_7446 | Fructose-bisphosphate aldolase | 1353.193 |
| FVE85_1309 | Profilin-2 | 1323.515 |
| FVE85_8054 | hypothetical protein FVE85_8054 | 1322.545 |
| FVE85_5088 | Peptidyl-prolyl cis-trans isomerase | 1303.259 |
| Genes in *italics* contain an intron and have not been considered for codon usage determination, promoters and 5’ UTRs of underlined genes have been used for promoter analysis. See Materials and methods for details. | | |

**Supplementary Table 3.** Plasmids and primers used for vector construction.

| **Vector** | **Construct** | **Vector backbone** | **Insert cloned** | **Insert amplified from** | **Primer sequence 5’-3’** | **Orientation** |
| --- | --- | --- | --- | --- | --- | --- |
| pASH1 | PpYFP | pZL22^1)^ | PpYFP, fully codon optimized *YFP* variant | synthesized gene | GAAGCAGATCGCAGCTCGAGGAGTGCAGGCAACatggtgtcgaagggcgag | for |
|  |  |  |  |  | GGCACTCTGAAGCCTTGTGAGAGCTAGCGCGACGTctacttgatcagctcgtccat | rev |
| pASH2 | cpYFP | pZL22^1)^ | cpYFP, *YFP* codon optimized for *Chlamydomonas* chloroplasts | pRMB8^2)^ | GAAGCAGATCGCAGCTCGAGGAGTGCAGGCAACATGGTTTCAAAAGGTGA | for |
|  |  |  |  |  | GGCACTCTGAAGCCTTGTGAGAGCTAGCGCGACGTTTATTTAATTAATTCATCCATACC | rev |
| pASH3 | vYFP | pZL22^1)^ | venus YFP (vYFP) | pJR39^2,3)^ | GAAGCAGATCGCAGCTCGAGGAGTGCAGGCAACATGGTGAGCAAGGGCGA | for |
|  |  |  |  |  | GGCACTCTGAAGCCTTGTGAGAGCTAGCGCGACGTTTACTTGATCAGCTCGTCCATGC | rev |
| pASH4 | laYFP | pZL22^1)^ | laYFP, lowly codon adapted for the *Chlamydomonas* nuclear genome | pRMB13^2)^ | GAAGCAGATCGCAGCTCGAGGAGTGCAGGCAACATGGTCTCGAAGGGGGAG | for |
|  |  |  |  |  | GGCACTCTGAAGCCTTGTGAGAGCTAGCGCGACGTTTACTTGATGAGCTCGTCCATCC | rev |
| pASH7 | CrYFP | pZL22^1)^ | CrYFP, codon optimized for the *Chlamydomonas* nuclear genome | pRMB12^2)^ | GAAGCAGATCGCAGCTCGAGGAGTGCAGGCAACATGGTGAGCAAGGGCGA | for |
|  |  |  |  |  | GGCACTCTGAAGCCTTGTGAGAGCTAGCGCGACGTTTACTTGATCAGCTCGTCCATGC | rev |
| pASH8 | - | pZL22^1)^ | insertion of a SpeI restriction site between promoter and CDS of *cat* | pRMB7^2)^ | GAAGCAGATCGCAGCTCGAGGAGTGCAGGCAACActagTATGGAGAAGAAGATCACCGGCTAC | for |
|  |  |  |  |  | GGCACTCTGAAGCCTTGTGAGAGCTAGCGCGACGTTTAGGCGCCGCCCTGCCA | rev |
| pASH45 | secYFP | pASH8 | PpYFP | synthetized gene | CTGATTGCAACAGTCCAGGCAGTGTCGAAGGGCGAGGAGCTG | for |
|  |  |  |  |  | GGCACTCTGAAGCCTTGTGAGAGCTAGCGCGACGTCTACTTGATCAGCTCGTCCAT | rev |
|  |  |  | carbonic anhydrase signal peptide FVE80_3079 | genomic DNA | GCAGCTCGAGGAGTGCAGGCAACActagtaTGCGTAAGATGACGCTCACGG | for |
|  |  |  |  |  | CAGCTCCTCGCCCTTCGACACTGCCTGGACTGTTGCAATCAG | rev |
| pASH48 | YFP-ER | pASH8 | PpYFP+HDEL | synthetized gene | CTGATTGCAACAGTCCAGGCAGTGTCGAAGGGCGAGGAGCTG | for |
|  |  |  |  |  | CTGAAGCCTTGTGAGAGCTAGCGCGACGTCTACAGCTCGTCGTGCTTGATCAGCTCGTCCATGCC | rev |
|  |  |  | carbonic anhydrase signal peptide FVE80_3079 | gDNA | GCAGCTCGAGGAGTGCAGGCAACActagtaTGCGTAAGATGACGCTCACGG | for |
|  |  |  |  |  | CAGCTCCTCGCCCTTCGACACTGCCTGGACTGTTGCAATCAG | rev |
| pASH42 | CA | pZL22^1)^ | carbonic anhydrase promoter (FVE85_3079) | gDNA | ACAGCTCCTCGCCCTTCGACACCATACTAGGGTTGCGTACGACGAACGTG | for |
|  |  |  |  |  | ACAGCTCCTCGCCCTTCGACACCATACTAGGGTTGCGTACGACGAACGTG | rev |
|  |  |  | PpYFP | pASH1 | CACGTTCGTCGTACGCAACCctagtatggtgtcgaagggcgaggagctgt | for |
|  |  |  |  |  | GGCACTCTGAAGCCTTGTGAGAGCTAGCGCGACGTctacttgatcagctcgtccat | rev |
| pASH43 | PsbQ | pZL22^1)^ | hypothetical protein (PsbQ) promoter FVE85_3577 | gDNA | CACGGAGTCGGACAATCTCGCACCGCAGATTGATGAGAAATCGGAGTAGC | for |
|  |  |  |  |  | CAGCTCCTCGCCCTTCGACACCATGCTAGACTTCTTACCTGTTAACAACTC | rev |
|  |  |  | PpYFP | pASH1 | GTTGTTAACAGGTAAGAAGTCTAGCATGGTGTCGAAGGGCGAG | for |
|  |  |  |  |  | GGCACTCTGAAGCCTTGTGAGAGCTAGCGCGACGTctacttgatcagctcgtccat | rev |
| pASH49 | Fo/Ni | pZL22^1)^ | putative transporter *YrhG* (formate/nitrite transporter) promoter FVE80_0107 | gDNA | CACGGAGTCGGACAATCTCGCACCGCTTTGCGCTCCACGATTCATG | for |
|  |  |  |  |  | ACAGCTCCTCGCCCTTCGACACCATTTTTCCTTGTTCCTTCTTGTCAAAC | rev |
|  |  |  | PpYFP | pASH1 | GGTTTGACAAGAAGGAACAAGGAAAAatggtgtcgaagggcgag | for |
|  |  |  |  |  | GGCACTCTGAAGCCTTGTGAGAGCTAGCGCGACGTctacttgatcagctcgtccat | rev |
| pASH50 | PYP1 | pZL22^1)^ | *PYP1* promoter FVE80_6364 | gDNA | CACGGAGTCGGACAATCTCGCACCGCGCGCGTTTCGTGCAAGC | for |
|  |  |  |  |  | ACAGCTCCTCGCCCTTCGACACCATACCCCCGTCACTTCCTCTC | rev |
|  |  |  | PpYFP | pASH1 | GGGAGAGGAAGTGACGGGGGTatggtgtcgaagggcgag | for |
|  |  |  |  |  | GGCACTCTGAAGCCTTGTGAGAGCTAGCGCGACGTctacttgatcagctcgtccat | rev |
| pASH52 | ChlBP | pZL22^1)^ | chlorophyll a/b-binding protein promoter FVE80_6435 | gDNA | CACGGAGTCGGACAATCTCGCACCGCCGAGTCATGAAGACTCTGTGATG | for |
|  |  |  |  |  | ACAGCTCCTCGCCCTTCGACACCATTTTGCTATCTGTGTGTGTCTGTTG | rev |
|  |  |  | PpYFP | pASH1 | CAACAGACACACACAGATAGCAAAatggtgtcgaagggcgag | for |
|  |  |  |  |  | GGCACTCTGAAGCCTTGTGAGAGCTAGCGCGACGTctacttgatcagctcgtccat | rev |
|  | | | | | | |
| 1) Li & Bock, 2018; 2) Barahimipour et al., 2015; 3) Neupert et al., 2009; for: forward primer; rev: reverse primer | | | | | | |
|  | | | | | | |

**Supplementary Table 4.** DNA sequences of all *YFP* gene variants (with their corresponding vector names) used in this study.

pASH1 (PpYFP):

ATGGTGTCGAAGGGCGAGGAGCTGTTCACGGGCGTGGTGCCGATCCTGGTGGAGCTGGACGGCGACGTGAACGGCCACAAGTTCTCGGTGTCGGGCGAGGGCGAGGGCGACGCGACGTACGGCAAGCTGACGCTGAAGCTGATCTGCACGACGGGCAAGCTGCCGGTGCCGTGGCCGACGCTGGTGACGACGCTGGGCTACGGCCTGCAGTGCTTCGCGCGCTACCCGGACCACATGAAGCAGCACGACTTCTTCAAGTCGGCGATGCCGGAGGGCTACGTGCAGGAGCGCACGATCTTCTTCAAGGACGACGGCAACTACAAGACGCGCGCGGAGGTGAAGTTCGAGGGCGACACGCTGGTGAACCGCATCGAGCTGAAGGGCATCGACTTCAAGGAGGACGGCAACATCCTGGGCCACAAGCTGGAGTACAACTACAACTCGCACAACGTGTACATCACGGCGGACAAGCAGAAGAACGGCATCAAGGCGAACTTCAAGATCCGCCACAACATCGAGGACGGCGGCGTGCAGCTGGCGGACCACTACCAGCAGAACACGCCGATCGGCGACGGCCCGGTGCTGCTGCCGGACAACCACTACCTGTCGTACCAGTCGGCGCTGTCGAAGGACCCGAACGAGAAGCGCGACCACATGGTGCTGCTGGAGTTCGTGACGGCGGCGGGCATCACGCTGGGCATGGACGAGCTGATCAAGTAG

pASH2 (cpYFP):

ATGGTTTCAAAAGGTGAAGAATTATTTACAGGTGTTGTTCCAATTTTAGTTGAATTAGATGGTGATGTAAATGGTCACAAATTTTCAGTATCAGGTGAAGGTGAAGGTGATGCTACATATGGTAAATTAACATTAAAATTAATTTGTACAACAGGTAAATTACCAGTTCCTTGGCCAACATTAGTTACAACATTAGGTTATGGTTTACAATGTTTTGCTCGTTATCCAGATCATATGAAACAACATGATTTTTTTAAATCAGCTATGCCAGAAGGTTATGTTCAAGAACGTACAATTTTTTTTAAAGATGATGGTAATTATAAAACACGTGCTGAAGTTAAATTTGAAGGTGATACATTAGTAAATCGTATTGAATTAAAAGGTATTGATTTTAAAGAAGATGGTAATATTTTAGGTCACAAATTAGAATATAATTATAATTCACACAATGTATATATTACAGCTGATAAACAAAAAAATGGTATTAAAGCTAATTTTAAAATTCGTCATAATATTGAAGATGGTGGTGTTCAATTAGCTGATCACTATCAACAAAATACACCAATTGGTGATGGTCCAGTATTATTACCAGATAATCATTATTTATCATATCAATCAGCTTTATCAAAAGATCCAAATGAAAAACGTGATCATATGGTATTATTAGAATTTGTAACAGCTGCTGGTATTACATTAGGTATGGATGAATTAATTAAATAA

pASH3 (vYFP):

ATGGTGAGCAAGGGCGAGGAGCTGTTCACCGGGGTGGTGCCCATCCTGGTCGAGCTGGACGGCGACGTAAACGGCCACAAGTTCAGCGTGTCCGGCGAGGGCGAGGGCGATGCCACCTACGGCAAGCTGACCCTGAAGCTGATCTGCACCACCGGCAAGCTGCCCGTGCCCTGGCCCACCCTCGTGACCACCCTGGGCTACGGCCTGCAGTGCTTCGCCCGCTACCCCGACCACATGAAGCAGCACGACTTCTTCAAGTCCGCCATGCCCGAAGGCTACGTCCAGGAGCGCACCATCTTCTTCAAGGACGACGGCAACTACAAGACCCGCGCCGAGGTGAAGTTCGAGGGCGACACCCTGGTGAACCGCATCGAGCTGAAGGGCATCGACTTCAAGGAGGACGGCAACATCCTGGGGCACAAGCTGGAGTACAACTACAACAGCCACAACGTCTATATCACCGCCGACAAGCAGAAGAACGGCATCAAGGCCAACTTCAAGATCCGCCACAACATCGAGGACGGCGGCGTGCAGCTCGCCGACCACTACCAGCAGAACACCCCCATCGGCGACGGCCCCGTGCTGCTGCCCGACAACCACTACCTGAGCTACCAGTCCGCCCTGAGCAAAGACCCCAACGAGAAGCGCGATCACATGGTCCTGCTGGAGTTCGTGACCGCCGCCGGGATCACTCTCGGCATGGACGAGCTGATCAAGTAA

pASH4 (laYFP):

ATGGTCTCGAAGGGGGAGGAGCTCTTCACGGGGGTCGTCCCGATCCTCGTCGAGCTCGACGGGGACGTCAACGGGCACAAGTTCTCGGTCTCGGGGGAGGGGGAGGGGGACGCGACGTACGGGAAGCTCACGCTCAAGCTCATCTGCACGACGGGGAAGCTCCCGGTCCCGTGGCCGACGCTCGTCACGACGCTCGGGTACGGGCTCCAGTGCTTCGCGCGGTACCCGGACCACATGAAGCAGCACGACTTCTTCAAGTCGGCGATGCCGGAGGGGTACGTCCAGGAGCGGACGATCTTCTTCAAGGACGACGGGAACTACAAGACGCGGGCGGAGGTCAAGTTCGAGGGGGACACGCTCGTCAACCGGATCGAGCTCAAGGGGATCGACTTCAAGGAGGACGGGAACATCCTCGGGCACAAGCTCGAGTACAACTACAACTCGCACAACGTCTACATCACGGCGGACAAGCAGAAGAACGGGATCAAGGCGAACTTCAAGATCCGGCACAACATCGAGGACGGGGGGGTCCAGCTCGCGGACCACTACCAGCAGAACACGCCGATCGGGGACGGGCCGGTCCTCCTCCCGGACAACCACTACCTCTCGTACCAGTCGGCGCTCTCGAAGGACCCGAACGAGAAGCGGGACCACATGGTCCTCCTCGAGTTCGTCACGGCGGCGGGGATCACGCTCGGGATGGACGAGCTCATCAAGTAA

pASH7 (CrYFP):

ATGGTGAGCAAGGGCGAGGAGCTGTTCACCGGCGTGGTGCCCATCCTGGTGGAGCTGGACGGCGACGTGAACGGCCACAAGTTCAGCGTGAGCGGCGAGGGCGAGGGCGACGCCACCTACGGCAAGCTGACCCTGAAGCTGATCTGCACCACCGGCAAGCTGCCCGTGCCCTGGCCCACCCTGGTGACCACCCTGGGCTACGGCCTGCAGTGCTTCGCCCGCTACCCCGACCACATGAAGCAGCACGACTTCTTCAAGAGCGCCATGCCCGAGGGCTACGTGCAGGAGCGCACCATCTTCTTCAAGGACGACGGCAACTACAAGACCCGCGCCGAGGTGAAGTTCGAGGGCGACACCCTGGTGAACCGCATCGAGCTGAAGGGCATCGACTTCAAGGAGGACGGCAACATCCTGGGCCACAAGCTGGAGTACAACTACAACAGCCACAACGTGTACATCACCGCCGACAAGCAGAAGAACGGCATCAAGGCCAACTTCAAGATCCGCCACAACATCGAGGACGGCGGCGTGCAGCTGGCCGACCACTACCAGCAGAACACCCCCATCGGCGACGGCCCCGTGCTGCTGCCCGACAACCACTACCTGAGCTACCAGAGCGCCCTGAGCAAGGACCCCAACGAGAAGCGCGACCACATGGTGCTGCTGGAGTTCGTGACCGCCGCCGGCATCACCCTGGGCATGGACGAGCTGATCAAGTAA

**Supplementary Table 5.** Quantification of YFP accumulation and total protein content per cell. A.U., arbitrary units; SD, standard deviation; Biol. repl., biological replicate.

Quantification of PpYFP (Figure 2b):

| **Standard** | |
| --- | --- |
| rGFP [ng] | A.U. |
| 5 | 1857 |
| 10 | 8283 |
| 25 | 25083 |
| 50 | 34771 |

| Sample | **Porphyridium** | | | | **Chlamydomonas** | |
| --- | --- | --- | --- | --- | --- | --- |
|  | PpYFP | | CrYFP | | CrYFP | |
|  | 1 | 2 | 1 | 2 | 1 | 2 |
| A.U. | 35244 | 36986 | 9659 | 8677 | 1594 | 5169 |
| ng/µg | 47.1 | 49.5 | 11.5 | 10.7 | 4.5 | 7.6 |
| % TSP | 4.71 | 4.95 | 1.15 | 1.07 | 0.45 | 0.76 |

Quantification of secYFP in the supernatant (Figure 7a):

|  | **secYFP in 50 µL supernatant** | | | | | |
| --- | --- | --- | --- | --- | --- | --- |
| Day | 3 | 4 | 5 | 6 | 7 | 10 |
| A.U | 28407 | 30730 | 41005 | 45609 | 49844 | 62392 |
| ng/50 µL | 27.76 | 31.39 | 47.45 | 54.65 | 61.27 | 80.89 |
| mg/L | 0.56 | 0.63 | 0.95 | 1.09 | 1.23 | 1.62 |

| **Standard** | |
| --- | --- |
| rGFP [ng] | A.U. |
| 5 | 5532 |
| 10 | 16627 |
| 25 | 27322 |
| 50 | 42379 |

Quantification of cytYFP (Figure 7b):

|  | **cytYFP (2 µg)** | | | | | |
| --- | --- | --- | --- | --- | --- | --- |
| Day | 3 | 4 | 5 | 6 | 7 | 10 |
| A.U | 36338 | 32950 | 30582 | 30286 | 30168 | 29711 |
| ng/2 µg | 48.48 | 42.71 | 38.67 | 38.16 | 37.96 | 37.18 |
| % of TP | 2.42 | 2.14 | 1.93 | 1.91 | 1.90 | 1.86 |

| **Standard** | |
| --- | --- |
| rGFP [ng] | A.U. |
| 5 | 5361 |
| 10 | 16748 |
| 25 | 27660 |
| 50 | 34631 |

Total protein content per cell:

|  |  | Protein concentration of the lysate [µg/µL] | | | | |  | Cells harvested |
| --- | --- | --- | --- | --- | --- | --- | --- | --- |
|  |  | Batch 1 | Batch 2 | Batch 3 | Batch 4 | Batch 5 |  | 1*10^7^ |
| Biol. repl. | A | 1.68 | 1.65 | 1.34 | 1.37 | 1.51 |  |  |
|  | B | 1.43 | 1.72 | 1.67 | 1.37 | 1.68 |  | Cells per µL protein lysate |
|  | C | 1.61 | 1.69 | 1.47 | 1.53 | 1.64 |  | 66666.67 |
|  |  |  |  |  |  |  |  |  |
| Protein/cell [pg] (n=15) | | | 23.36 |  |  |  |  |  |
| SD | | | 2.23 |  |  |  |  |  |


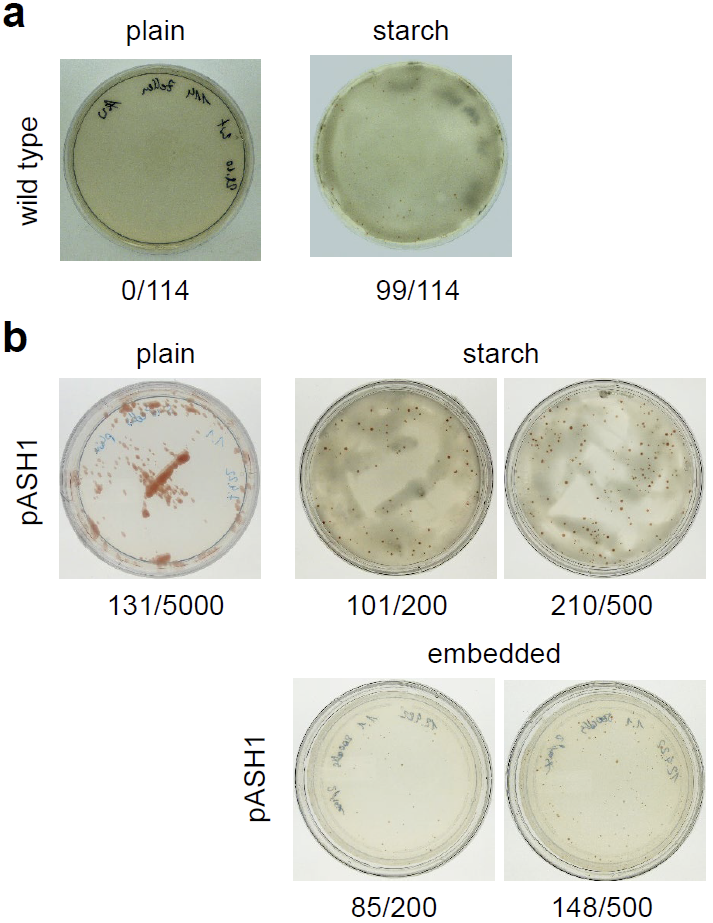


**Supplementary Figure 1.** Efficiency of colony formation from single cells with different plating methods. (**a**) A dilution equivalent to 114 wild-type cells was either plated directly on lsASW agar plates (plain), or mixed with 1.2 mL starch suspension (0.5 g/mL) and then plated on lsASW agar plates (starch). No formation of colonies was visible after two weeks of incubation under continuous illumination (100 µmol photons m^-2^ s^-1^ light intensity) upon direct plating, whereas approximately 86% of the cells were recovered as colonies with the starch plating method. (**b**) A transgenic strain was plated with different methods on (zeocin-containing) lsASW agar plates, and the efficacy of colony recovery was calculated. The directly plated cells (plain) formed a local lawn (presumably due to cell aggregation) and showed only a low number of distinct colonies. The starch method yielded an efficiency of colony formation of approximately 46%, and the embedding method, where plated cells are embedded in a thin film of 0.4% agar on top of the lsASW agar yielded an efficiency of colony formation of around 36%.


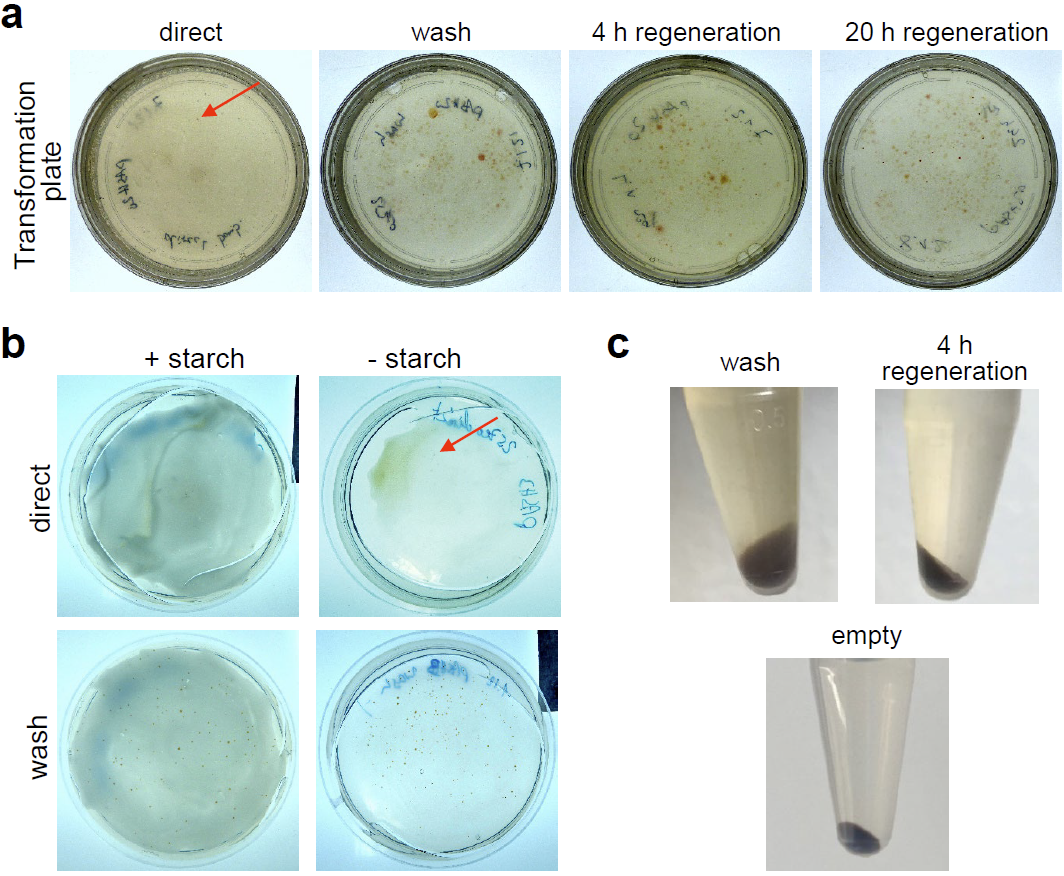


**Supplementary Figure 2.** Development of an improved transformation protocol for *Porphyridium purpureum*. (**a**) Comparison of the previously used transformation protocol (~~#11809{[~~Li and Bock, 2018~~]}~~) with the new protocol. In the previously established protocol, *Porphyridium* cells are directly plated on zeocin-containing lsASW plates, bombarded with plasmid vector-coated gold particles and regenerated on the same plate (labeled “direct”). In the new protocol, cells are bombarded on lsASW plates without antibiotics, then washed off the plates, washed twice with lsASW, and then plated either directly on zeocin-containing plates (wash), or after a recovery period in liquid medium of 4 or 20 hours (“4 h regeneration” and “20 h regeneration”, respectively). Note that the wash alone is sufficient to greatly increase the number of colonies forming after 3 weeks. (**b**) The same protocol was also tested for cells plated with starch, to possibly further support colony formation. While the starch had no significant influence on colony formation after transformation, the wash substantially increased the number of transformants recovered. (**c**) Supernatant after the first wash with lsASW following biolistic bombardment without a recovery phase (“wash”) and after a recovery phase of 4 h (“4 h regeneration”). The greenish color of the supernatant indicates broken cells that are washed away by the procedure. As a control, pelleted unbombarded cells are shown (“empty”).


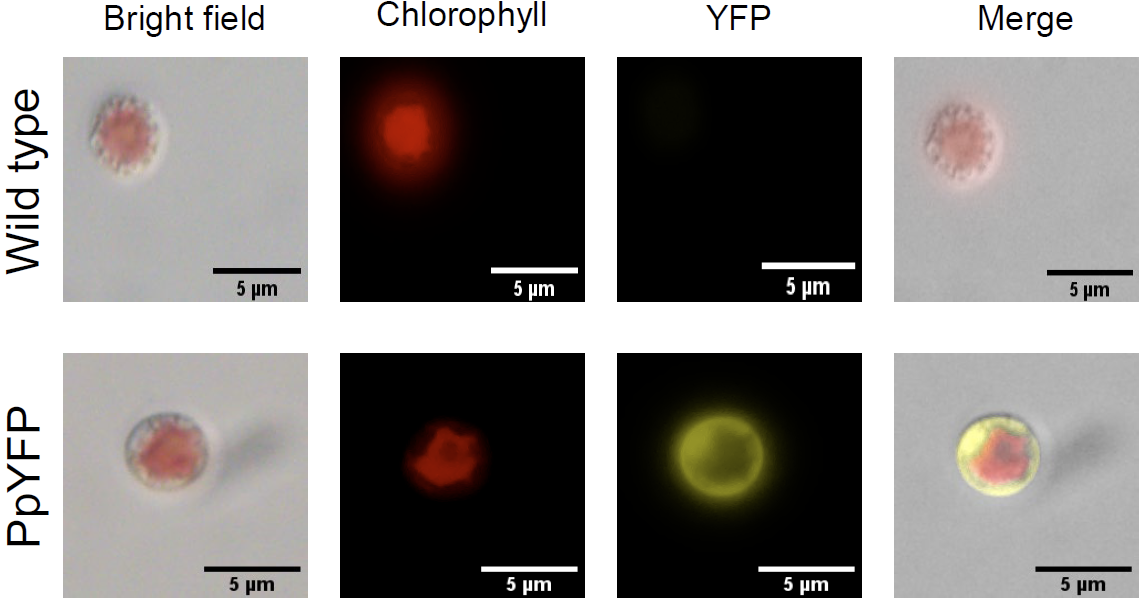


**Supplementary Figure 3.** Subcellular localization of the cytosolic PpYFP in a *Porphyridium purpureum* cell. The red chlorophyll fluorescence and the merged image show the single plastid present in *P. purpureum* cells. A wild-type cell is shown as negative control.


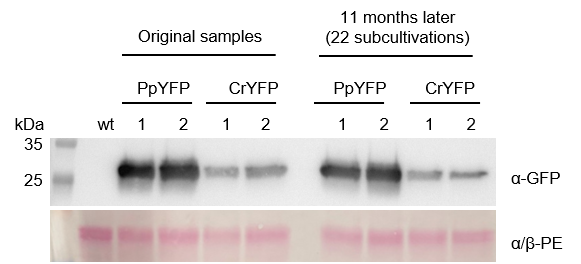


**Supplementary Figure 4.** Long-term stability of transgene expression in the transgenic algal strains. 5 µg of total protein from *PpYFP* and *CrYFP* transformants extracted directly after their isolation (original samples, see Figure 2) and after 11 months of subcultivation in the presence of zeocin (equivalent to 22 cultivation rounds) were loaded. Very similar protein accumulation levels confirm the stability of YFP expression and plasmid maintenance over time. Relevant bands of the molecular weight marker are labeled (with the sizes in kDa).
